# Numerical studies on a ternary AgInTe_2_ chalcopyrite thin film solar cell

**DOI:** 10.1016/j.heliyon.2023.e19011

**Published:** 2023-08-07

**Authors:** Arifuzzaman Joy, Ahnaf Tahmid Abir, Bipanko Kumar Mondal, Jaker Hossain

**Affiliations:** a Solar Energy Laboratory, Department of Electrical and Electronic Engineering, University of Rajshahi, Rajshahi 6205, Bangladesh; b Department of Electrical & Electronic Engineering, Pundra University of Science & Technology, Bogura, Bogura 5800, Bangladesh

**Keywords:** AgInTe_2_, AlSb, BaSi_2_, Thin film solar cell, SCAPS-1D

## Abstract

This paper theoretically outlines a new *n*-AlSb/*p*-AgInTe_2_/*p*^*+*^-BaSi_2_ solar cell. The dominance of several factors such as depth, carrier density and defects of every layer on the photovoltaic (PV) outcome has been ascertained applying Solar Cell Capacitance Simulator (SCAPS)-1D computer-based simulator. The AgInTe_2_ (AIT) solar cell has been probed for finding the role of BaSi_2_ as a back surface field (BSF) layer. It is revealed that the device power conversion efficiency (PCE) increments from 30% to 34% owing to the use of BaSi_2_ semiconducting BSF with V_OC_ = 0.90 V, J_SC_ = 43.75 mA/cm^2^, FF = 86.42%, respectively. The rippling of the output parameters with respect to the change in series and shunt resistances has also been probed and demonstrated. All the findings reveal the prospect of *n*-AlSb/*p*-AIT/*p*^*+*^-BaSi_2_ dual-heterojunction thin film photovoltaic cell.

## Highlights

• AgInTe_2_ chalcopyrite thin film solar cell has been designed and simulated with SCAPS-1D
• *n*-AlSb/*p*-AgInTe_2_ thin film solar cell shows a PCE of 30%
• BaSi_2_ BSF layer enhances PCE to 34.32% with V_OC_ = 0.90 V, J_SC_ = 43.75 mA/cm^2^ and FF = 86.42%
• *n*-AlSb/*p*-AIT/*p*^*+*^ -BaSi_2_ dual-heterojunction thin film solar cell shows its future potential

## Graphical abstract

**Figure:**
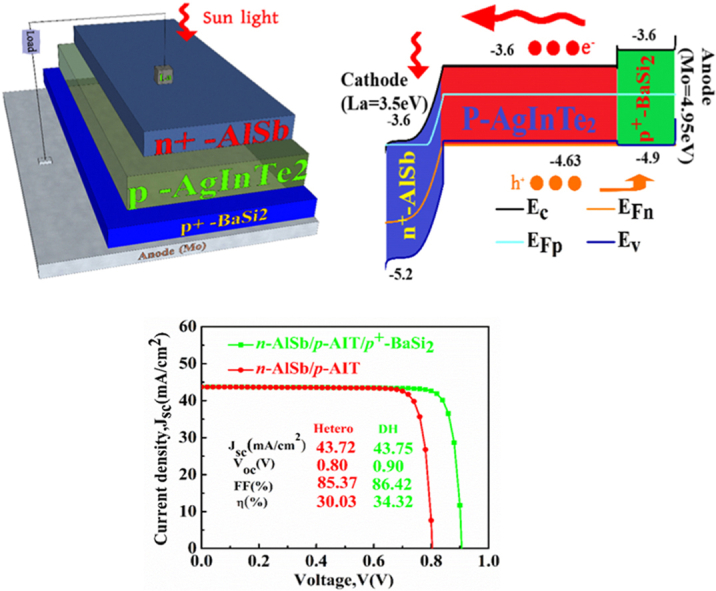

## 1 Introduction

Strong demand for renewable energy has grown in recent years and even the most biomass fuel producing countries are intending to use renewable energy to feed their energy hungriness. Therefore, the world needs high efficiency solar cells as renewable energy sources. Solar fuel can also stand as an alternative to fossil fuel which use diverse photocatalysts in environmentally friendly way [1]. However, the obstacles that are arising in the playground of massive production of high efficiency solar cells are the high production cost, both in terms of manufacturing and recycling end-of-life cells [2]. In addition, there has been difficulty in developing larger sized cells that can be integrated economically into existing solar panel formats. Besides, at low temperature they exhibit deficiency in stability as well as some perovskite solar cells are made of detrimental component such as lead (Pb) [3]. Each of these technical issues has played a role in slowing the market penetration of solar cells. Silicon-based photovoltaic (PV) industries are striving to minimize module fabrication price by commencing a combination of thin wafers along with boosting cell efficiency. To elevate the efficiency the cell, the silicon wafer width has been lessened lower than 100 μm, by virtue of the progress in open circuit voltage (V_OC_) of the PV cell with a finite Auger recombination [[4], [5], [6], [7], [8]]. In the year of 2017, Kaneka Corporation has manifested a highly efficient silicon heterojunction (HJ) photovoltaic cell with a record PCE of 26.7% and the research has opened a new path in the area of solar photovoltaic cell [9]. However, the theoretical efficiency limit for a single-heterojunction solar cell is 29.4% and this efficiency is just above 2.7% from that reported by Kaneka [10]. Therefore, emergency of a novel method has arisen as this is very close to the performance limit. Dual-heterojunction (DH) solar cells have been proposed so that the efficiency of solar cells could be ameliorated [11]. In case of dual-heterojunction solar cell, the Shockley–Queisser (SQ) efficiency boundary is 42–46% [12,13]. Therefore, there is a scope for the efficiency enhancement through DH structure.

Herein, a ternary alloy AgInTe_2_-based thin film solar cell has been studied for high efficiency. AgInTe_2_ (AIT) is one of the I-III-VI_2_ triune chalcopyrite mixture which has got a special animus because of its application to photovoltaic solar cells and optical devices [14,15]. Some researchers have focused on AgInTe_2_ and the majority of which belongs to elastic constants and specific heat [16]. However, only a few papers have presented the optical and electrical properties of AIT [17]. The AIT has a straight bandgap and it is at the Γ point [[18], [19], [20], [21]], moreover, the density of states (DOS) is steep, which leads to a large Seebeck coefficient [22]. For this reason, AgInTe_2_ may be proved as a p-type thermoelectric materials with a doping in the range of 10^19^–10^20^ cm^−3^. The thermoelectric transport characteristic confides both on the temperature and on the doping concentration [23].

AgInTe_2_ is really novel in the field of photovoltaics and has been used as the absorber layer only in a few works. So far, a couple of reports reveal AIT solar cell with AgInTe_2_/In_2_S_3_/TiO_2_/FTO structure where AIT has been deposited by printing and RF sputtering deposition methods and Au has been used as an electrode [24,25]. The efficiency has been reported in the range of 0.5–1.13%. The efficiency is low mainly due to the lower V_OC_ and FF which may results from the inappropriate choice of window layer and also deposition method plays important role in high quality film deposition. However, AIT is capable to prove itself as a perfect absorber layer because of obtaining some quality of an ideal absorption layer, for an instance, enriched crystallographic properties, suitable carrier lifetime, exalted optical absorption coefficient and lofty mobility [26].

In addition, a window layer or buffer layer is a layer which stands just over the absorber layer and doped with the opposite conductive material. The window layer is generally used to build a pn junction in a heterojunction thin film solar cell with the absorber layer [27]. An exalted bandgap, pony thickness, and humble series resistance are expected with the window layer for aerial optical throughput. In the composition of a solar cell, window layer material provides a fateful job to enhance the efficiency of a solar cell [28]. Aluminium antimonide (AlSb) could be a spanking option as a window layer in AIT-based thin film solar cell. AlSb is a part of group III-V material having a bandgap of 1.6 eV at a temperature of 300 K [29]. Moreover, AlSb has some other features to choose it as the window material, such as its high melting and boiling point of 1330 and 2740 K, respectively. The most important parameter of AlSb is its index of refraction of 3.3 at 200 nm wavelength, and dielectric constant is 10.9 at radiowave frequencies [30]. Moreover, various technics are available for the deposition of AlSb thin films for an instance hot wall epitaxy, co-evaporation and co-sputtering etc. [31]. However, AlSb has yet not been used with AgInTe_2_ based solar cell.

The back surface field (BSF) is a heavily doped layer with a doping of the same type as that of the absorber layer to obtain the pp^+^ structure. With the help of BSF layer, it is possible to enlarge the short circuit current, the spectral response and the curtailment of contract resistance. Due to the difference between the doping level of the absorber and BSF layers, a potential barrier is generated which try to incarcerate the minority carriers in the absorber layer [32]. Barium Silicide (BaSi_2_) has been used as a BSF layer in this AIT-based thin film solar cell. BaSi_2_ is a sanguine material for enormously efficient thin-film heterostructure solar cell [33]. BaSi_2_ is likeable in photovoltaic application for its lofty durability and bandgap of approximately 1.1–1.35 eV [34]. There is an affluence of both Ba and Si in the earth, as a result the BaSi_2_ can be used to make a cheap dual-heterojunction solar cell [35]. BaSi_2_ is fabricated with the high purity Ge (HPGe) thin film fabrication technique, Vapor phase epitaxy (VPE) technique, molecular beam epitaxy (MBE) technique, solid phase epitaxy (SPE) technique etc. [[36], [37], [38], [39]]. Besides, there is another method called magnetron sputtering method (MSM) which is held on the radio frequency (RF) for developing polycrystalline BaSi_2_ films at a subordinate cost on glass substrate [40]. The most exciting characteristics for which BaSi_2_ can be used as the BSF layer are the high absorption coefficient of about 3 × 10^5^ cm^−1^, a standard indirect bandgap, the diffusion distance of 10 μm, and the minority carrier lifetime of 14 μs [41]. However, as far as we know, there are no records available depicting the usage of BaSi_2_ as the BSF layer with AIT-based solar cell.

In this endeavor, we present a novel AIT-based double-heterojunction (DH) thin film photovoltaic cell. Herein, AlSb, AgInTe_2_ and BaSi_2_ have been utilized as the *n*-window, *p*-absorber and *p*^*+*^-BSF layers, respectively. The *n*-AlSb/*p*-AgInTe_2_/*p*^*+*^-BaSi_2_ devices have been evaluated to get superior output Photovoltaic (PV) performances with computational simulations. The quantum efficiency (QE) of the photovoltaic device has also been enumerated and delimitated in niceties with output photovoltaic parameters such as J_SC_, V_OC_, FF and efficiency. This work premises that the AIT-based solar cell with AlSb as window and BaSi_2_ as BSF may get high importance in the upcoming days.

## 2 Device architecture and numerical computation

**Fig. 1:**
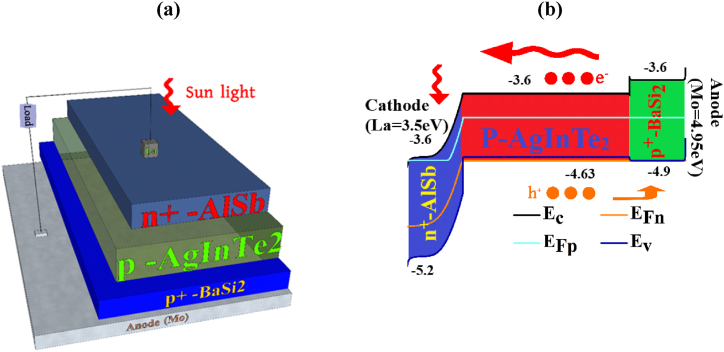
The (a) Designed architecture, (b) electronic energy diagram of *n*-AlSb/*p*-AgInTe_2_/*p*^*+*^*-*BaSi_2_ thin film solar cell.

Fig. 1(a) delimitates the schematic diagram of the presented AgInTe_2_ chalcopyrite-based dual-heterojunction solar cell and the energy band diagram is delineated in Fig. 1(b). AgInTe_2_ is a *p*-type material with an optical bandgap of 1.03 eV, electron affinity of 3.6 eV, and ionization energy of 4.63 eV which has been used as a solar absorber layer. It is capable to form a pn heterojunction with the *n*-type AlSb material, which has a bandgap of 1.6 eV and an electron affinity of 3.6 eV. With these identical values, they form a suitable *n*-AlSb/*p*-AgInTe_2_ heterojunction. On the opposite side, the BaSi_2_ which has seized a bandgap of 1.3 eV and electron affinity of 3.3 eV is susceptible to form a pp ^+^ heterojunction with AgInTe_2_ material. So, three of them in association have made a congruous *n*-AlSb/*p*-AIT/*p*^*+*^-BaSi_2_ heterojunction solar cell. The light enters the cell through the *n*-AlSb window layer of the device. Moreover, Lanthanum with a work function of 3.5 eV and molybdenum with a work function of 4.95 eV have been utilized as the hindmost and foremost contract, respectively for efficient charge collection.

The proposed device structure was simulated by SCAPS 1D simulator (version 3.3.07), delivered from Professor M. Burgelman and his group, University of Gent, Belgium, which essentially resolves Poisson's equations of continuity for holes and electrons. The simulation was done under 1 sun irradiation with a power density of 100 mW/cm^2^ of global air mass (AM) of 1.5G spectrum. The absorption coefficient data for the AgInTe_2_ absorber, the BaSi_2_ BSF, and the AlSb window layer were assigned from the SCAPS traditional E_g_-sqrt model with default values. Defects have a significant impact on how well a solar cell performs. In the simulation, donor/acceptor/acceptor type of defects were set for window/absorber/BSF layer, respectively. Gaussian shaped energetic distribution was used for the defects in all layers with default capture cross-section for electrons and holes. This simulation avoided radiative recombination and auger recombination as large number of bulk defects were considered. The surface-recombination velocity of electron/hole that affects the quantum efficiency and reverse saturation current was set to 10^5^/10^7^ cm/s for the front and 10^7^/10^5^ cm/s for back metallic contacts. The physical parameters of different layers were taken from reported works as shown in Table 1.

**Table 1:** The various parameters of AlSb, AIT and BaSi_2_ layers put in the calculation of *n*-AlSb/*p*-AIT/*p*^*+*^*-*BaSi_2_ thin film solar cell.

Parameters	*n*-AlSb [42,43]	*p*-AgInTe_2_ [44,45]	*p*^*+*^-BaSi_2_ [46,47]
Bandgap (eV)	1.6	1.03	1.30
Electron affinity	3.6	3.6	3.3
Thickness (μm)	0.2	0.6	0.2
Dielectric permittivity (relative)	12.04	8.9	10
Effective DOS at CB (cm^−3^)	7.8 × 10^17^	3.66 × 10^19^	1.0 × 10^19^
Effective DOS at VB (cm^−3^)	1.8 × 10^19^	1.35 × 10^19^	1.0 × 10^19^
Electron thermal velocity (cm/s)	1.7 × 10^7^	1.0 × 10^7^	1.0 × 10^7^
Hole thermal velocity (cm/s)	1.4 × 10^7^	1.0 × 10^7^	1.0 × 10^7^
Hole mobility (cm^2^/vs)	4.2 × 10^2^	8.870 × 10^2^	2.0 × 10^1^
Electron mobility (cm^2^/vs)	2 × 10^2^	1.011 × 10^3^	2.0 × 10^1^
Shallow uniform donor density, N_D_ (cm^−3^)	1 × 10^17^	0	0
Shallow uniform acceptor density, N_A_ (cm^−3^)	0	1.0 × 10^20^	1.0 × 10^20^
Bulk defects (cm^−3^)	1 × 10^14^	1 × 10^13^	1 × 10^14^

Defects at various interfaces:	
Heterointerfaces	Defect density (cm^−2^)
*n*^*+*^ -AlSb/*p*-AgInTe_2_	1.00 × 10^10^
*p*-AgInTe_2_/*p*^*+*^-BaSi_2_	1.00 × 10^10^

## 3 Results and discussion

The output parameters of a photovoltaic (PV) cell for example short circuit current density (J_SC_), open circuit voltage (V_OC_), fill factor (FF), and efficiency (η) vary with the thicknesses of different layers, for an instance the window, absorber, and back surface field (BSF) layer, and with the carrier concentration and defect density of those layers. The performance parameters also vary with the shunt and series resistances, which depend on temperature. The maximum output of the AIT solar cell has been found from optimizing the device structure.

### 3.1 Device outcome with AIT absorber layer

In this part, the influences of AIT semiconducting layer on PV parameters of *n-*AlSb/*p-*AgInTe_2_/*p*^*+*^*-*BaSi_2_ solar cell have been studied. The depth, doping density, and defect density of the absorber layer have been varied from 0.25 to 1.5 μm, 1 × 10^17^ to 1 × 10^22^ cm^−3^, and 1 × 10^11^ to 1 × 10^16^ cm^−3^, respectively. The width, doping concentration, and volume defects of the window and the back surface field layers have been kept fixed as shown in Table 1.

**Fig. 2:**
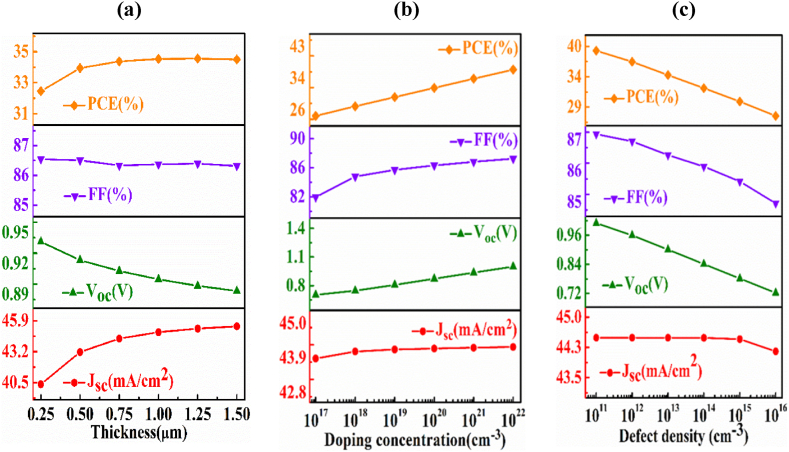
The fluctuation of output performance parameters (Voc, Jsc, FF, ƞ) of *n-*AlSb/*p-*AgInTe_2_/*p*^*+*^*-*BaSi_2_ photovoltaic cell as a function of (a) thickness, (b) carrier and (c) defects of AgInTe_2_ absorber layer.

Fig. 2(a) delineates the photovoltaic output parameters of *n-*AlSb/*p-*AIT/*p*^*+*^*-*BaSi_2_ solar cell with varying the breadth of the absorber layer. It is visualized in the figure that both the fill factor and open-circuit voltage decrease with mounting width of the absorber layer and both the short circuit current and efficiency (η) increase with increasing thickness. The J_SC_ and η of the device increase from 40.5 to 45.9 mA/cm^2^ and from 33 to 35%, respectively. The thicker absorber layer enhances the possibility of more light absorption. As a result, more electron and hole pairs are created which enhances the short circuit current [48]. On the opposite site, as the reverse saturation current enhances in accordance to the thickness, there is a negative change on the value of V_OC_ from 0.95 to 0.89 V and FF from 87 to 86% [49]. However, the power conversion efficiency (PCE) of the device increases depending on the significant increase of J_SC_.

Fig. 2(b) presents the dependence of the photovoltaic parameters of *n-*AlSb/*p-*AgInTe_2_/*p*^*+*^*-*BaSi_2_ solar cell on the fluctuation of the doping concentration of the AIT absorber layer. The carrier density of the layer enrolled to absorb photons has a fateful role on the PV parameters. The expression which relates the V_OC_ with carrier concentration is V_OC_ =(kT/q) ln [(N_A_ + Δn)Δn/n_i_^2^], where, n_i_ stands for intrinsic concentration, doping concentration is denoted by N_A_ and excess carrier is denoted by Δn [50]. It can be noticed that all performance parameters increase with increasing carrier in the AIT absorber layer. This is because, with the advancement of doping concentration, the mobility of carrier also increase which leads to increase in short circuit current [51]. Concurrently, the value of V_OC_ rises from 0.8 to 1 V as with the increment of hole density which results from the rise in built-in voltage with doping. Moreover, there is a crucial change on the value of the fill factor and the efficiency from 82 to 86% and 26% to 34%, respectively. The reason behind this is amelioration of doping concentration degrades the value of series resistance [52].

Fig. 2(c) presents the reliance of the PV output performance of *n-*AlSb/*p-*AgInTe_2_/*p*^*+*^*-*BaSi_2_ photovoltaic cell on the modulation of the defects of the AIT layer. All performance parameters are seen to decline with growing density of defect of the absorber layer except the grade of J_SC_ which has maintained almost a constant value up to 10^15^ cm^−3^. Beyond this boundary, the value of J_SC_ depicts a decrement. The grade of V_OC_, FF and PCE depict a change from 1 to 0.72 V, 87 to 84.5% and 40 to 29%, respectively. This is because defects can raise the reverse saturation current and decrease the mobility of carriers [53]. The PCE of the device reaches to 40% when defect density is fixed at 1 × 10^11^ and further increasing of defect density makes down of efficiency.

### 3.2 Device outcome with AlSb window layer

To investigate the dependency of the AlSb window layer, the width, doping concentration, and defect density of the AlSb layer have been altered from 0.1 to 0.6 μm, 1 × 10^15^ to 1 × 10^20^ cm^−3^, and 1 × 10^11^ to 1 × 10^16^ cm^−3^, respectively.

**Fig. 3:**
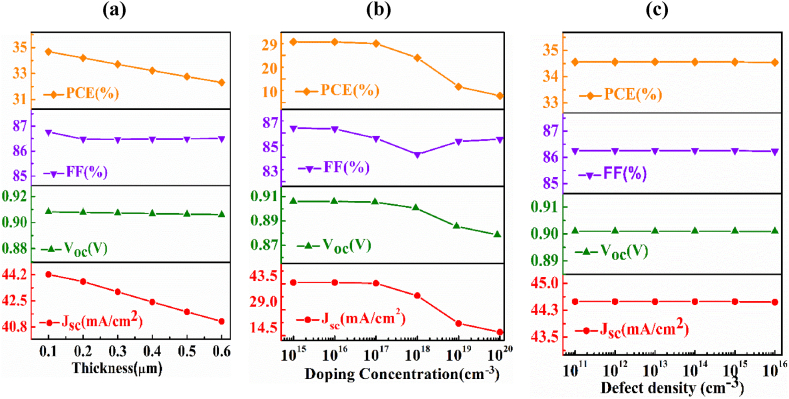
The fluctuation of output performance (Voc, Jsc, FF, ƞ) of *n-*AlSb/*p-*AIT/*p*^*+*^*-*BaSi_2_ solar PV cell as a function of (a) thickness, (b) carrier density and (c) defects of AlSb window layer.

Fig. 3(a) delineates the consequence of the variation of the window layer's thickness on the PV parameters of the *n-*AlSb/*p-*AgInTe_2_/*p*^*+*^*-*BaSi_2_ solar cell. The J_SC_ and PCE are noticed to decrease with the ameliorating of the thickness of the AlSb layer, this is because of the enhancement of parasitic absorption which prevents the photons from having a lower wavelength to approach the absorber layer [54]. The boundary of the alternation of J_SC_ is from 44 to 40 mA/cm^2^. The maximum efficiency of 35% is obtained at an initial thickness of 0.1 μm, then it reduces to 31% at 0.6 μm width. On the contrary, the value of V_OC_ and FF are not affected much by the fluctuation of the width of the window layer. For the reason of high carrier mobility in association with a wide bandgap, the depth of the window layer cannot manipulate the PV parameters strongly [55].

The influence of doping concentration of AlSb window on the PV outcomes of *n-*AlSb/*p-*AgInTe_2_/*p*^*+*^*-*BaSi_2_ solar cell has been depicted in Fig. 3(b). It is seen that the J_SC_ and efficiency are very sensitive to the doping concentration and their values follow a downward direction. In the studied range of carrier concentration, the J_SC_ and PCE decrease from 43.5 to 14.5 mA/cm^2^ and from 29 to 10%, respectively, though the V_OC_ and FF are not so sensitive to the carrier concentration. The increase in free carrier recombination at greater doping concentrations is what has caused the decrease in J_SC_ and PCE [56].

Fig. 3(c) shows the change in PV performances of the *n-*AlSb/*p-*AgInTe_2_/*p*^*+*^*-*BaSi_2_ device with the defect density of the AlSb layer. It is noted that the defects up to 10^16^ cm^−3^ in the AlSb layer have almost no impact on the output PV parameters of the AIT photovoltaic device. However, further increment of defect density increases the dark current which may have a serious influence on device performances [57].

Hence, it can be concluded that the AlSb window layer could be used to control the optical losses and the electrical peculiarity of the *n-*AlSb/*p-*AIT/*p*^*+*^*-*BaSi_2_ thin film PV device.

### 3.3 Device outcome with BaSi_2_ back surface field layer

In this part, the effect of BaSi_2_ BSF layer on the *n-*AlSb/*p-*AgInTe_2_/*p*^*+*^*-*BaSi_2_ photovoltaic device has been probed in detail. The width, doping density, and defect density of the BaSi_2_ BSF layer have been altered from 0.1 to 0.6 μm, 1 × 10^17^ to 1 × 10^22^ cm^−3^, and 1 × 10^11^ to 1 × 10^16^ cm^−3^, respectively.

**Fig. 4:**
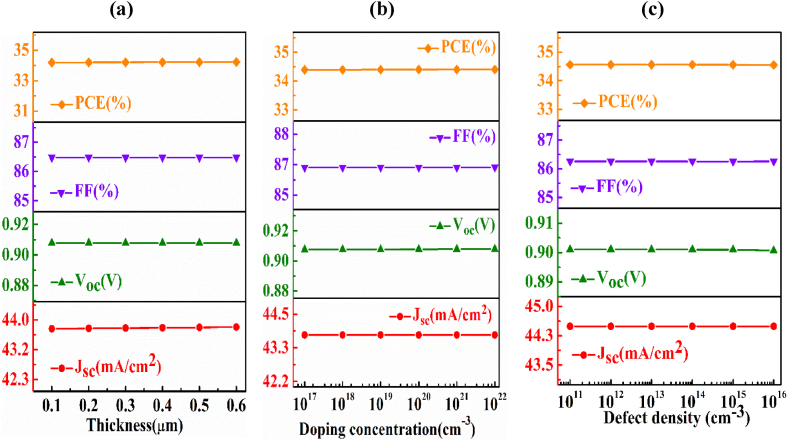
The fluctuation of output performance (Voc, Jsc, FF, ƞ) of *n-*AlSb/*p-*AgInTe_2_/*p*^*+*^*-*BaSi_2_ device as a function of (a) thickness, (b) carrier and (c) defect density of BaSi_2_ back surface layer.

Fig. 4(a) describes the dominance of BSF layer breadth on the photovoltaic outcomes of the *n-*AlSb/*p-*AgInTe_2_/*p*^*+*^*-*BaSi_2_ PV cell and no variations of the output parameters have been found with respect to the alternation of thickness. But, further advancement of thickness of BaSi_2_ may have a negative role on the PV parameters. The cause behind this is with the improvement of BSF thickness the series resistance is also enhanced [58].

Fig. 4(b) displays how the PV parameters are related to the alteration of the doping concentration of the BSF layer. No change has been recorded throughout the observed range but it may be predicted that beyond this range there is a slight negation of the PV parameters. The auger recombination may get domination with a higher doping concentration which is harmful to efficiency [59].

Fig. 4(c) imprints the character of the fluctuation of the defects in the BaSi_2_ BSF layer on the PV output of the presented solar PV cell. With the improvement of defect density, there is only a weeny change in the performance parameters has been noticed which can be considered to be constant. But the access amount of defects may have advanced the dark current which is dangerous for the activity of the presented solar cell [57].

### 3.4 Impact of resistances on device performance

The photovoltaic performance of a cell massively influenced by the series and shunt resistances of the device. The sources of these resistances are the attachment among various active layers, metal connections, and defects related to fabrication [60,61]. The series resistance is liable for causing a drop of voltage across the body of the PV device, whereas the shunt resistance makes a short path in the device for the current when the applied voltage across the cell is zero.

**Fig. 5:**
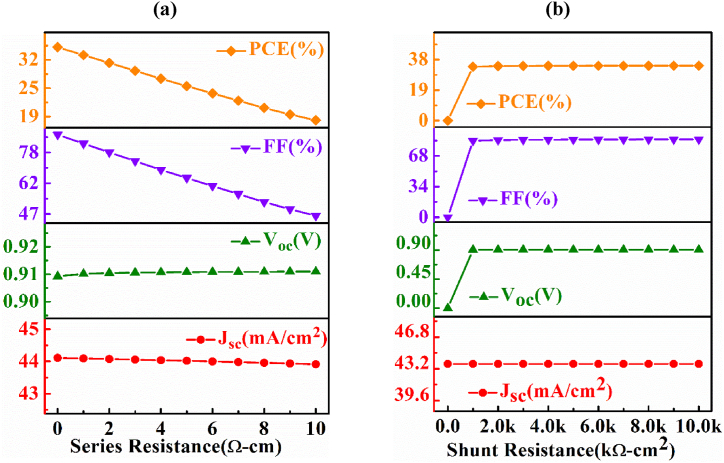
The impact of (a) series and (b) shunt resistances on *n-*AlSb/*p-*AgInTe_2_/*p*^*+*^*-*BaSi_2_ solar cell.

Fig. 5(a) shows the modulation of output parameters with the switching of series resistance. It is perceived that both the V_OC_ and J_SC_ are less sensitive to the variation of series resistance but both the FF and efficiency are highly sensitive to the change of series resistance. This is because the improvement of series resistance decreases the FF tremendously.

Fig. 5(b) sketches the alteration of output parameters of the *n-*AlSb/*p-*AgInTe_2_/*p*^*+*^*-*BaSi_2_ DH PV device as a function of shunt resistance. Except J_SC_, all the output parameters show a positive change with the improvement of shunt resistance up to a value of 1.5 kΩ/cm^2^ and then they stay at a constant value. In other words, with the decrement of the shunt resistance the performance parameters delineate a negative impression. Thus the highest value of PCE should be recorded with the lowest value of series resistance and the highest value of shunt resistance.

### 3.5 QE with and without BaSi_2_ BSF layer

**Fig. 6:**
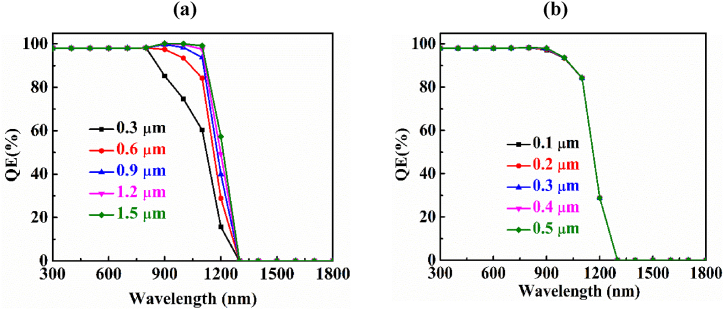
Changing of quantum efficiency as a function of wavelength for (a) AIT absorber and (b) BaSi_2_ BSF layer at various thicknesses.

The ratio between the total aggregated charges to the quantity of alit photons is denoted as the quantum efficiency (QE) of a photovoltaic cell. We get the maximum QE of 100% when all the incident photons are converted into electric charges. We measure the quantum efficiency as a function of wavelength [53,57]. In Fig. 6(a), the quantum efficiency has been depicted in regard to the fluctuation of wavelength with deferent thicknesses of the absorber layer. This graph shows that the quantum efficiency escalates with the rising of the absorber layer thickness. The reason behind this is the absorption of photons enhanced with a wider absorber layer which also makes more electron-hole pairs resulting higher J_SC_.

Fig. 6(b) displays the undulation of quantum efficiency with different thickness of the BaSi_2_ BSF layer. No variation of quantum efficiency is recorded with the seesaw of the width of the BSF layer just because the thickness of back surface field (BSF) is not creating the barrier for charge carriers. The BSF helps to confine the minority carriers generated at the surface of the PV cell. If the BSF is too thin, the minority carriers will diffuse out of the solar cell before they can be collected by the electrodes. If the BSF is too thick, the minority carriers will have a difficult time reaching the electrodes. The thickness of the BSF is therefore critical to the performance of the solar cell.

### 3.6 Overall output of *n*-AlSb/*p*-AIT/*p*^*+*^-BaSi_2_ photovoltaic cell

**Fig. 7:**
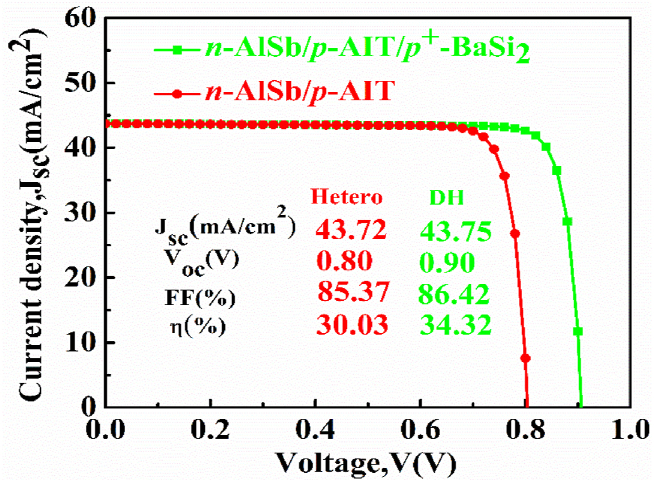
The Current (J)-Voltage (V) characteristics of the AIT solar cell including and excluding BaSi_2_ BSF layer.

Herein, the contribution of the *n*-AlSb/*p*-AIT/*p*^*+*^-BaSi_2_ photovoltaic cell has been analyzed. Fig. 7 shows the current (J)-voltage (V) graphs of the *n*-AlSb/*p*-AIT heterojunction and *n*-AlSb/*p*-AIT/*p*^*+*^-BaSi_2_ double-heterojunction PV devices with fine-tune structures. The optimal thicknesses of AlSb window, AIT absorber and BaSi_2_ semiconductors are 0.2, 0.6, and 0.2 μm, orderly. The doping concentration of the same layers are 10^17^, 10^20^, and 10^20^ cm^−3^, accordingly. However, the defect densities are fixed at 10^14^, 10^13^ and 10^14^ cm^−3^ for window, absorber and BSF layers, respectively. It is visualized from the figure that the *n*-AlSb/*p*-AIT cell architecture attains output parameters J_SC_ = 43.72 mA/cm^2^, V_OC_ = 0.80 V, FF = 85.37% and efficiency = 30.03%. Whereas, with the inclusion of BaSi_2_ layer, the *n*-AlSb/*p*-AIT heterojunction device turns into *n*-AlSb/*p*-AIT/*p*^*+*^-BaSi_2_ PV device and the there is an improvement on the output parameters. The improved output parameters are J_SC_ = 43.75 mA/cm^2^, V_OC_ = 0.90 V, FF = 86.42% and efficiency = 34.32%. The J_SC_ experiences only a meager change as all the alit photons are absorbed in AIT layer before reaching the relatively wide bandgap BaSi_2_ BSF layer. However, there is a noticeable rise in the value of V_OC_ as the cause of the improvement of supreme built-in voltage at the *n*-AlSb/*p*-AIT and *p*-AIT/*p*^*+*^-BaSi_2_ heterojunctions. An improvement in the efficiency is also noticed and the reason behind this is the advancement of the value of V_OC_.

However, in order to validate the potential of BaSi_2_ BSF layer, CuInSe_2_ semiconductor based CdS/*p*-CuInSe_2_/*p*^*+*^-BaSi_2_ solar cell has been considered. CuInSe_2_ (CIS) is an I-III-VI group material like AIT with an optical bandgap of 1.04 eV which is close to that of AIT (1.03 eV) [62]. CIS based ZnO/*n*-CdS/*p*-CIS with Mo metal as anode shows an experimental efficiency of ∼15% with a V_OC_ of 0.513 V, J_SC_ = 40.40 mA/cm^2^ and FF = 71.6% [63]. The same structure has been used in SCAPS that produces an efficiency of 16.39% with V_OC_ = 0.526 V, J_SC_ = 39.65 mA/cm^2^, and FF = 78.58% which is almost consistent with the experimental result. Then, BaSi_2_ BSF layer has been added in the structure with the physical parameters shown in Table 1. The ZnO/*n*-CdS/*p*-CIS/*p*^*+*^-BaSi_2_ device with Mo anode provides a PCE of 21.65% with a V_OC_ = 0.624 V, J_SC_ = 45.25 mA/cm^2^ and FF = 76.06%. Therefore, it can be concluded that as BaSi_2_ BSF layer shows potential in CuInSe_2_ based ZnO/n-CdS/p-CuInSe_2_/*p*^+^-BaSi_2_ device, it will also have high impact on *n*-AlSb/*p*-AgInTe_2_/*p*^*+*^-BaSi_2_ dual-heterojunction thin film photovoltaic cells.

## 4 Conclusion

In this effort, we have explored the operation of a photovoltaic device based on AgInTe_2_ ternary chalcopyrite semiconducting material. AgInTe_2_ as the absorber layer, AlSb as the window layer, and BaSi_2_ as the back surface field layer have been chosen for the device structure. Best performance has been attained by taking the absorber width of 0.6 μm, BSF layer thickness of 0.2 μm and the window layer thickness of 0.2 μm. The excellent values of performance parameters which have been obtained are J_SC_ = 43.75 mA/cm^2^, V_OC_ = 0.90 V, FF = 86.42% and efficiency = 34.32%. Undoubtedly, these numbers are rich in the present days. Further research in this device may attain more fruitful results in future. Hopefully, these outcomes instigate the potential of highly effective AIT-based *n*-AlSb/*p*-AIT/*p*^*+*^-BaSi_2_ photovoltaic cell to combat the world energy crisis.

## Author contribution statement

Arifuzzaman Joy: Ahnaf Tahmid Abir: Jaker Hossain: Conceived and designed the experiments; Performed the experiments; Analyzed and interpreted the data; Contributed reagents, materials, analysis tools or data; Wrote the paper.

Bipanko Kumar Mondal: Analyzed and interpreted the data; Wrote the paper.

## Data availability statement

Data will be made available on request.

## Declaration of competing interest

The authors declare that they have no known competing financial interests or personal relationships that could have appeared to influence the work reported in this paper.
