# Supplementary material for: Numerical studies on a ternary AgInTe2 chalcopyrite thin film solar cell
Source: Heliyon. 2023 Aug 7;9(8):e19011. doi: 10.1016/j.heliyon.2023.e19011 (PMC10432989; doi:10.1016/j.heliyon.2023.e19011)
Supplement: Multimedia component 1 [file mmc1.pdf]

Numerical studies on a ternary AgInTe2 chalcopyrite thin film solar cell

By Jaker Hossain

WORD COUNT	6288	TIME SUBMITTED	15-JUL-2023 11:20AM
		PAPER ID	101255709

Numerical studies on a ternary $AgInTe_2$ chalcopyrite thin film solar cell

Arifuzzaman Joy[1], Ahnaf Tahmid Abir[1], Bipanko Kumar Mondal[1,2], and Jaker Hossain[1*]

[1]*Solar Energy Laboratory, Department of Electrical and Electronic Engineering, University of Rajshahi, Rajshahi 6205, Bangladesh.*

[2]*Department of Electrical & Electronic Engineering, Pundra University of Science & Technology, Bogura, Bogura 5800, Bangladesh.*

Abstract

This paper theoretically outlines a new *n*-AlSb/*p*-$AgInTe_2$/p^+-$BaSi_2$ solar cell. The dominance of several factors such as depth, carrier density and defects of every layer on the photovoltaic (PV) outcome has been ascertained applying Solar Cell Capacitance Simulator (SCAPS)-1D computer-based simulator. The $AgInTe_2$ (AIT) solar cell has been probed for finding the role of $BaSi_2$ as a back surface field (BSF) layer. It is revealed that the device power conversion efficiency (PCE) increments from 30% to 34% owing to the use of $BaSi_2$ semiconducting BSF with V_{OC}=0.90 V, J_{SC}=43.75 mA/cm^2, FF=86.42%, respectively. The rippling of the output parameters with respect to the change in series and shunt resistances has also been probed and demonstrated. All the findings reveal the prospect of *n*-AlSb/*p*-AIT/p^+-$BaSi_2$ dual-heterojunction thin film photovoltaic cell.

Keywords: $AgInTe_2$, AlSb, $BaSi_2$, thin film solar cell, SCAPS-1D.

1. Introduction

Strong demand for renewable energy has grown in recent years and even the most biomass fuel producing countries are intending to use renewable energy to feed their energy hungriness. Therefore, the world needs high efficiency solar cells as renewable energy sources. Solar fuel can

also stand as an alternative to fossil fuel which use diverse photocatalysts in environmentally friendly way [1]. However, the obstacles that are arising in the playground of massive production of high efficiency solar cells are the high production cost, both in terms of manufacturing and recycling end-of-life cells [2]. In addition, there has been difficulty in developing larger sized cells that can be integrated economically into existing solar panel formats. Besides, at low temperature they exhibit deficiency in stability as well as some perovskite solar cells are made of detrimental component such as lead (Pb) [3]. Each of these technical issues has played a role in slowing the market penetration of solar cells. Silicon-based photovoltaic (PV) industries are striving to minimize module fabrication price by commencing a combination of thin wafers along with boosting cell efficiency. To elevate the efficiency the cell, the silicon wafer width has been lessened lower than 100 µm, by virtue of the progress in open circuit voltage (V_{OC}) of the PV cell with a finite Auger recombination [4-8]. In the year of 2017, Kaneka Corporation has manifested a highly efficient silicon heterojunction (HJ) photovoltaic cell with a record PCE of 26.7% and the research has opened a new path in the area of solar photovoltaic cell [9]. However, the theoretical efficiency limit for a single-heterojunction solar cell is 29.4% and this efficiency is just above 2.7% from that reported by Kaneka [10]. Therefore, emergency of a novel method has arisen as this is very close to the performance limit. Dual-heterojunction (DH) solar cells have been proposed so that the efficiency of solar cells could be ameliorated [11]. In case of dual-heterojunction solar cell, the Shockley–Queisser (SQ) efficiency boundary is 42–46% [12-13]. Therefore, there is a scope for the efficiency enhancement through DH structure.

Herein, a ternary alloy $AgInTe_2$-based thin film solar cell has been studied for high efficiency. $AgInTe_2$ (AIT) is one of the I-III-VI_2 triune chalcopyrite mixture which has got a special animus because of its application to photovoltaic solar cells and optical devices [14-15]. Some researchers have focused on $AgInTe_2$ and the majority of which belongs to elastic constants and specific heat [16]. However, only a few papers have presented the optical and electrical properties of AIT [17]. The AIT has a straight bandgap and it is at the Γ point [18-21], moreover, the density of states (DOS) is steep, which leads to a large Seebeck coefficient [22]. For this reason, $AgInTe_2$ may be proved as a p-type thermoelectric materials with a doping in the range of 10^{19}–10^{20} cm^{-3}. The thermoelectric transport characteristic confides both on the temperature and on the doping concentration [23].

$AgInTe_2$ is really novel in the field of photovoltaics and has been used as the absorber layer only in a few works. So far, a couple of reports reveal AIT solar cell with $AgInTe_2/In_2S_3/TiO_2$/FTO structure where AIT has been deposited by printing and RF sputtering deposition methods and Au has been used as an electrode [24-25]. The efficiency has been reported in the range of 0.5-1.13%. The efficiency is low mainly due to the lower V_{OC} and FF which may results from the inappropriate choice of window layer and also deposition method plays important role in high quality film deposition. However, AIT is capable to prove itself as a perfect absorber layer because of obtaining some quality of an ideal absorption layer, for an instance, enriched crystallographic properties, suitable carrier lifetime, exalted optical absorption coefficient and lofty mobility [26].

In addition, a window layer or buffer layer is a layer which stands just over the absorber layer and doped with the opposite conductive material. The window layer is generally used to build a pn junction in a heterojunction thin film solar cell with the absorber layer [27]. An exalted bandgap, pony thickness, and humble series resistance are expected with the window layer for aerial optical throughput. In the composition of a solar cell, window layer material provides a fateful job to enhance the efficiency of a solar cell [28]. Aluminium antimonide (AlSb) could be a spanking option as a window layer in AIT-based thin film solar cell. AlSb is a part of group III-V material having a bandgap of 1.6 eV at a temperature of 300 K [29]. Moreover, AlSb has some other features to choose it as the window material, such as its high melting and boiling point of 1330 and 2740 K, respectively. The most important parameter of AlSb is its index of refraction of 3.3 at 200 nm wavelength, and dielectric constant is 10.9 at radiowave frequencies [30]. Moreover, various technics are available for the deposition of AlSb thin films for an instance hot wall epitaxy, co-evaporation and co-sputtering etc. [31]. However, AlSb has yet not been used with $AgInTe_2$ based solar cell.

The back surface field (BSF) is a heavily doped layer with a doping of the same type as that of the absorber layer to obtain the pp^+ structure. With the help of BSF layer, it is possible to enlarge the short circuit current, the spectral response and the curtailment of contract resistance. Due to the difference between the doping level of the absorber and BSF layers, a potential barrier is generated which try to incarcerate the minority carriers in the absorber layer [32]. Barium Silicide ($BaSi_2$) has been used as a BSF layer in this AIT-based thin film solar cell. $BaSi_2$ is a sanguine material for enormously efficient thin-film heterostructure solar cell [33]. $BaSi_2$ is likeable in photovoltaic

application for its lofty durability and bandgap of approximately 1.1-1.35 eV [34]. There is an affluence of both Ba and Si in the earth, as a result the $BaSi_2$ can be used to make a cheap dual-heterojunction solar cell [35]. $BaSi_2$ is fabricated with the high purity Ge (HPGe) thin film fabrication technique, Vapor phase epitaxy (VPE) technique, molecular beam epitaxy (MBE) technique, solid phase epitaxy (SPE) technique etc. [36-39]. Besides, there is another method called magnetron sputtering method (MSM) which is held on the radio frequency (RF) for developing polycrystalline $BaSi_2$ films at a subordinate cost on glass substrate [40]. The most exciting characteristics for which $BaSi_2$ can be used as the BSF layer are the high absorption coefficient of about 3×10^5 cm^{-1}, a standard indirect bandgap, the diffusion distance of 10 μm, and the minority carrier lifetime of 14 μs [41]. However, as far as we know, there are no records available depicting the usage of $BaSi_2$ as the BSF layer with AIT-based solar cell.

In this endeavor, we present a novel AIT-based double-heterojunction (DH) thin film photovoltaic cell. Herein, AlSb, $AgInTe_2$ and $BaSi_2$ have been utilized as the n-window, p-absorber and p^+-BSF layers, respectively. The n-AlSb/p-$AgInTe_2$/p^+-$BaSi_2$ devices have been evaluated to get superior output Photovoltaic (PV) performances with computational simulations. The quantum efficiency (QE) of the photovoltaic device has also been enumerated and delimitated in niceties with output photovoltaic parameters such as J_{SC}, V_{OC}, FF and efficiency. This work premises that the AIT-based solar cell with AlSb as window and $BaSi_2$ as BSF may get high importance in the upcoming days.

2. Device architecture and numerical computation

Fig. 1(a) delimitates the schematic diagram of the presented $AgInTe_2$ chalcopyrite-based dual-heterojunction solar cell and the energy band diagram is delineated in Fig. 1(b). $AgInTe_2$ is a p-type material with an optical bandgap of 1.03 eV, electron affinity of 3.6 eV, and ionization energy of 4.63 eV which has been used as a solar absorber layer. It is capable to form a pn heterojunction with the n-type AlSb material, which has a bandgap of 1.6 eV and an electron affinity of 3.6 eV. With these identical values, they form a suitable n-AlSb/p-$AgInTe_2$ heterojunction. On the opposite side, the $BaSi_2$ which has seized a bandgap of 1.3 eV and electron affinity of 3.3 eV is susceptible to form a pp^+ heterojunction with $AgInTe_2$ material. So, three of them in association have made a congruous n-AlSb/p-AIT/p^+-$BaSi_2$ heterojunction solar cell. The light enters the cell

through the n-AlSb window layer of the device. Moreover, Lanthanum with a work function of 3.5 eV and molybdenum with a work function of 4.95 eV have been utilized as the hindmost and foremost contract, respectively for efficient charge collection.

The proposed device structure was simulated by SCAPS 1D simulator (version 3.3.07), delivered from Professor M. Burgelman and his group, University of Gent, Belgium, which essentially resolves Poisson's equations of continuity for holes and electrons. The simulation was done under 1 sun irradiation with a power density of 100 mW/cm^2 of global air mass (AM) of 1.5G spectrum. The absorption coefficient data for the $AgInTe_2$ absorber, the $BaSi_2$ BSF, and the AlSb window layer were assigned from the SCAPS traditional E_g-sqrt model with default values. Defects have a significant impact on how well a solar cell performs. In the simulation, donor/acceptor/acceptor type of defects were set for window/absorber/BSF layer, respectively. Gaussian shaped energetic distribution was used for the defects in all layers with default capture cross-section for electrons and holes. This simulation avoided radiative recombination and auger recombination as large number of bulk defects were considered. The surface-recombination velocity of electron/hole that affects the quantum efficiency and reverse saturation current was set to $10^5/10^7$ cm/s for the front and $10^7/10^5$ cm/s for back metallic contacts. The physical parameters of different layers were taken from reported works as shown in Table 1.

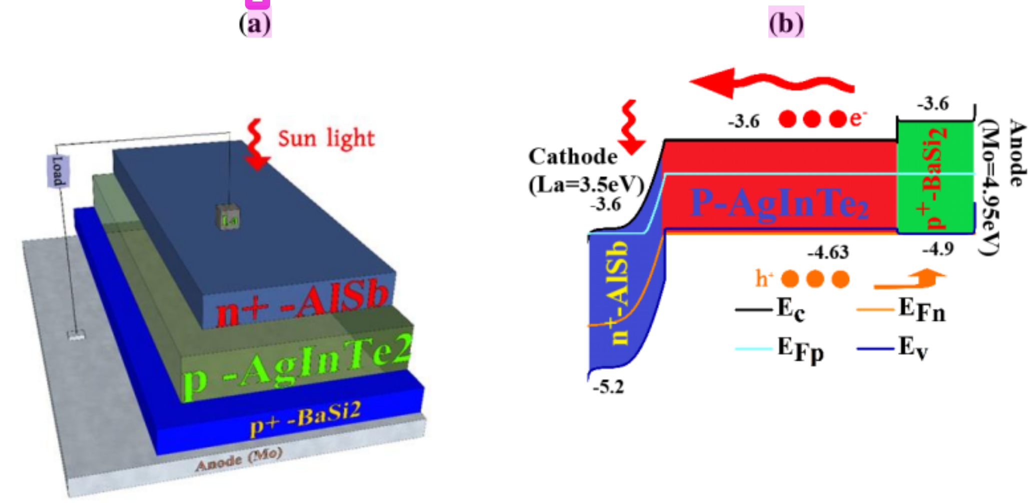

Fig. 1: The (a) Designed architecture, (b) electronic energy diagram of n-AlSb/p-$AgInTe_2$/p^+-$BaSi_2$ thin film solar cell.

Table 1: The various parameters of AlSb, AIT and $BaSi_2$ layers put in the calculation of *n*-AlSb/*p*-AIT/p^+-$BaSi_2$ thin film solar cell.

**Parameters**	***n*-AlSb [42-43]**	***p*-$AgInTe_2$[44-45]**	**p^+-$BaSi_2$ [46-47]**
Bandgap (eV)	1.6	1.03	1.30
Electron affinity	3.6	3.6	3.3
Thickness (μm)	0.2	0.6	0.2
Dielectric permittivity(relative)	12.04	8.9	10
Effective DOS at CB (cm^{-3})	7.8×10^{17}	3.66×10^{19}	1.0×10^{19}
Effective DOS at VB (cm^{-3})	1.8×10^{19}	1.35×10^{19}	1.0×10^{19}
Electron thermal velocity (cm/s)	1.7×10^{7}	1.0×10^{7}	1.0×10^{7}
Hole thermal velocity (cm/s)	1.4×10^{7}	1.0×10^{7}	1.0×10^{7}
Hole mobility (cm^2/vs)	4.2×10^{2}	8.870×10^{2}	2.0×10^{1}
Electron mobility (cm^2/vs)	2×10^{2}	1.011×10^{3}	2.0×10^{1}
Shallow uniform donor density, N_D(cm^{-3})	1×10^{17}	0	0
Shallow uniform acceptor density, N_A(cm^{-3})	0	1.0×10^{20}	1.0×10^{20}
Bulk defects (cm^{-3})	1×10^{14}	1×10^{13}	1×10^{14}
**Defects at various interfaces:**			
Heterointerfaces	Defect density (cm^{-2})		
n^+-AlSb/*p*-$AgInTe_2$	1.00×10^{10}		
p-$AgInTe_2$/p^+ -$BaSi_2$	1.00×10^{10}		

3. Results and discussion

The output parameters of a photovoltaic (PV) cell for example short circuit current density (J_{SC}), open circuit voltage (V_{OC}), fill factor (FF), and efficiency (η) vary with the thicknesses of different layers, for an instance the window, absorber, and back surface field (BSF) layer, and with the carrier concentration and defect density of those layers. The performance parameters also vary with the shunt and series resistances, which depend on temperature. The maximum output of the AIT solar cell has been found from optimizing the device structure.

3.1 Device outcome With AIT absorber layer

In this part, the influences of AIT semiconducting layer on PV parameters of *n*-AlSb/*p*-$AgInTe_2$/p^+-$BaSi_2$ solar cell have been studied. The depth, doping density, and defect density of the absorber layer have been varied from 0.25 to 1.5 μm, 1×10^{17} to 1×10^{22} cm^{-3} and 1×10^{11} to 1×10^{16} cm^{-3}, respectively. The width, doping concentration, and volume defects of the window and the back surface field layers have been kept fixed as shown in Table 1.

Fig. 2(a) delineates the photovoltaic output parameters of *n*-AlSb/*p*-AIT/p^+-$BaSi_2$ solar cell with varying the breadth of the absorber layer. It is visualized in the figure that both the fill factor and open-circuit voltage decrease with mounting width of the absorber layer and both the short circuit current and efficiency (η) increase with increasing thickness. The J_{SC} and η of the device increase from 40.5 to 45.9 mA/cm^2 and from 33 to 35%, respectively. The thicker absorber layer enhances the possibility of more light absorption. As a result, more electron and hole pairs are created which enhances the short circuit current [48]. On the opposite site, as the reverse saturation current enhances in accordance to the thickness, there is a negative change on the value of V_{OC} from 0.95 to 0.89 V and FF from 87 to 86% [49]. However, the power conversion efficiency (PCE) of the device increases depending on the significant increase of J_{SC}.

Fig. 2(b) presents the dependence of the photovoltaic parameters of *n*-AlSb/*p*-$AgInTe_2$/p^+-$BaSi_2$ solar cell on the fluctuation of the doping concentration of the AIT absorber layer. The carrier density of the layer enrolled to absorb photons has a fateful role on the PV parameters. The expression which relates the V_{OC} with carrier concentration is $V_{OC} =(kT/q) \ln [(N_A + \Delta n)\Delta n/ n_i^2]$, where, n_i stands for intrinsic concentration, doping concentration is denoted by N_A and excess carrier is denoted by Δn [50]. It can be noticed that all performance parameters increase with increasing carrier in the AIT absorber layer. This is because, with the advancement of doping

concentration, the mobility of carrier also increase which leads to increase in short circuit current [51]. Concurrently, the value of V_{OC} rises from 0.8 to 1 V as with the increment of hole density which results from the rise in built-in voltage with doping. Moreover, there is a crucial change on the value of the fill factor and the efficiency from 82 to 86% and 26% to 34%, respectively. The reason behind this is amelioration of doping concentration degrades the value of series resistance [52].

Fig. 2(c) presents the reliance of the PV output performance of *n*-AlSb/*p*-$AgInTe_2$/*p*⁺-$BaSi_2$ photovoltaic cell on the modulation of the defects of the AIT layer. All performance parameters are seen to decline with growing density of defect of the absorber layer except the grade of J_{SC} which has maintained almost a constant value up to 10^{15} cm^{-3}. Beyond this boundary, the value of J_{SC} depicts a decrement. The grade of V_{OC}, FF and PCE depict a change from 1 to 0.72 V, 87 to 84.5% and 40 to 29%, respectively. This is because defects can raise the reverse saturation current and decrease the mobility of carriers [53]. The PCE of the device reaches to 40% when defect density is fixed at 1×10^{11} and further increasing of defect density makes down of efficiency.

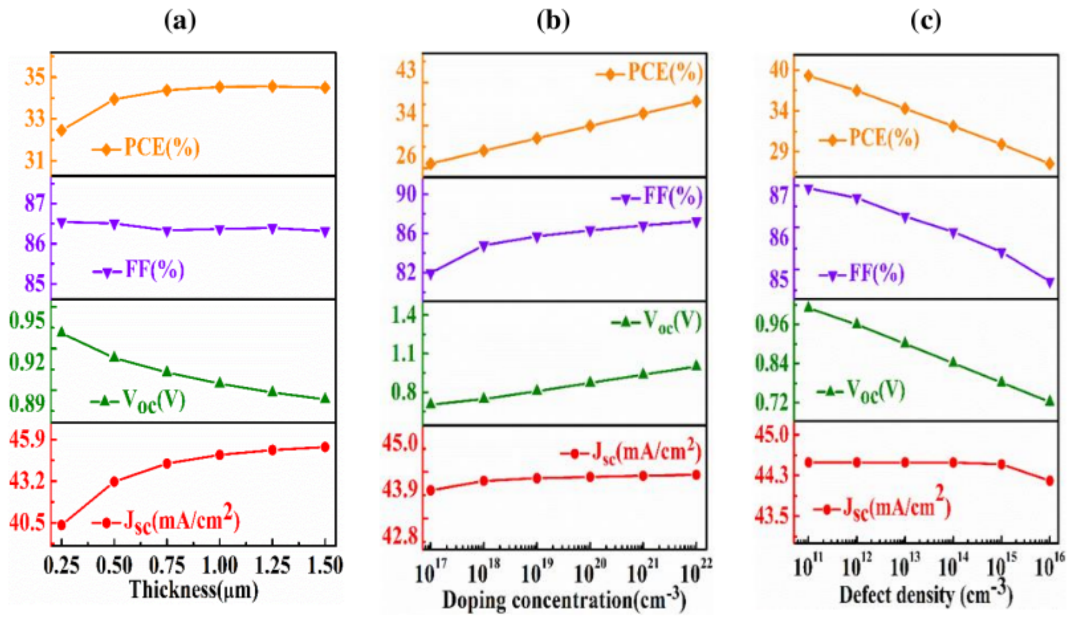

Fig. 2: The fluctuation of output performance parameters (Voc, Jsc, FF, η) of *n*-AlSb/*p*-$AgInTe_2$/*p*⁺-$BaSi_2$ photovoltaic cell as a function of (a) thickness, (b) carrier and (c) defects of $AgInTe_2$ absorber layer.

3.2 Device outcome with AlSb window layer

To investigate the dependency of the AlSb window layer, the width, doping concentration, and defect density of the AlSb layer have been altered from 0.1 to 0.6 μm, 1×10^{15} to 1×10^{20} cm^{-3}, and 1×10^{11} to 1×10^{16} cm^{-3}, respectively.

Fig. 3(a) delineates the consequence of the variation of the window layer's thickness on the PV parameters of the *n*-AlSb/*p*-$AgInTe_2$/p^+-$BaSi_2$ solar cell. The J_{SC} and PCE are noticed to decrease with the ameliorating of the thickness of the AlSb layer, this is because of the enhancement of parasitic absorption which prevents the photons from having a lower wavelength to approach the absorber layer [54]. The boundary of the alternation of J_{SC} is from 44 to 40 mA/cm^2. The maximum efficiency of 35% is obtained at an initial thickness of 0.1 μm, then it reduces to 31% at 0.6 μm width. On the contrary, the value of V_{OC} and FF are not affected much by the fluctuation of the width of the window layer. For the reason of high carrier mobility in association with a wide bandgap, the depth of the window layer cannot manipulate the PV parameters strongly [55].

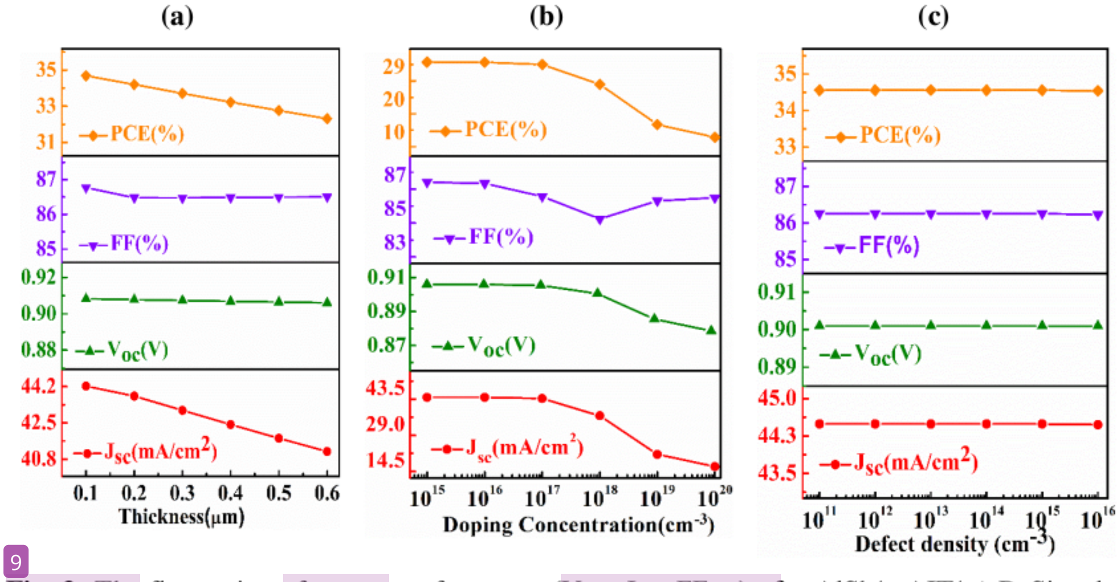

Fig. 3: The fluctuation of output performance (V_{oc}, J_{sc}, FF, η) of *n*-AlSb/*p*-AIT/p^+-$BaSi_2$ solar PV cell as a function of (a) thickness, (b) carrier density and (c) defects of AlSb window layer.

The influence of doping concentration of AlSb window on the PV outcomes of *n*-AlSb/*p*-$AgInTe_2$/p^+-$BaSi_2$ solar cell has been depicted in Fig. 3(b). It is seen that the J_{sc} and efficiency are very sensitive to the doping concentration and their values follow a downward direction. In the studied range of carrier concentration, the J_{SC} and PCE decrease from 43.5 to 14.5 mA/cm^2 and

from 29 to 10%, respectively, though the V_{OC} and FF are not so sensitive to the carrier concentration. The increase in free carrier recombination at greater doping concentrations is what has caused the decrease in J_{SC} and PCE [56].

Fig. 3(c) shows the change in PV performances of the *n*-AlSb/*p*-$AgInTe_2$/p^+-$BaSi_2$ device with the defect density of the AlSb layer. It is noted that the defects up to 10^{16} cm^{-3} in the AlSb layer have almost no impact on the output PV parameters of the AIT photovoltaic device. However, further increment of defect density increases the dark current which may have a serious influence on device performances [57].

Hence, it can be concluded that the AlSb window layer could be used to control the optical losses and the electrical peculiarity of the *n*-AlSb/*p*-AIT/p^+-$BaSi_2$ thin film PV device.

3.4 Device outcome with $BaSi_2$ back surface field layer

In this part, the effect of $BaSi_2$ BSF layer on the *n*-AlSb/*p*-$AgInTe_2$/p^+-$BaSi_2$ photovoltaic device has been probed in detail. The width, doping density, and defect density of the $BaSi_2$ BSF layer have been altered from 0.1 to 0.6 μm, 1×10^{17} to 1×10^{22} cm^{-3}, and 1×10^{11} to 1×10^{16} cm^{-3}, respectively.

Fig. 4(a) describes the dominance of BSF layer breadth on the photovoltaic outcomes of the *n*-AlSb/*p*-$AgInTe_2$/p^+-$BaSi_2$ PV cell and no variations of the output parameters have been found with respect to the alternation of thickness. But, further advancement of thickness of $BaSi_2$ may have a negative role on the PV parameters. The cause behind this is with the improvement of BSF thickness the series resistance is also enhanced [58].

Fig. 4(b) displays how the PV parameters are related to the alteration of the doping concentration of the BSF layer. No change has been recorded throughout the observed range but it may be predicted that beyond this range there is a slight negation of the PV parameters. The auger recombination may get domination with a higher doping concentration which is harmful to efficiency [59].

Fig. 4(c) imprints the character of the fluctuation of the defects in the $BaSi_2$ BSF layer on the PV output of the presented solar PV cell. With the improvement of defect density, there is only a weeny change in the performance parameters has been noticed which can be considered to be

constant. But the access amount of defects may have advanced the dark current which is dangerous for the activity of the presented solar cell [57].

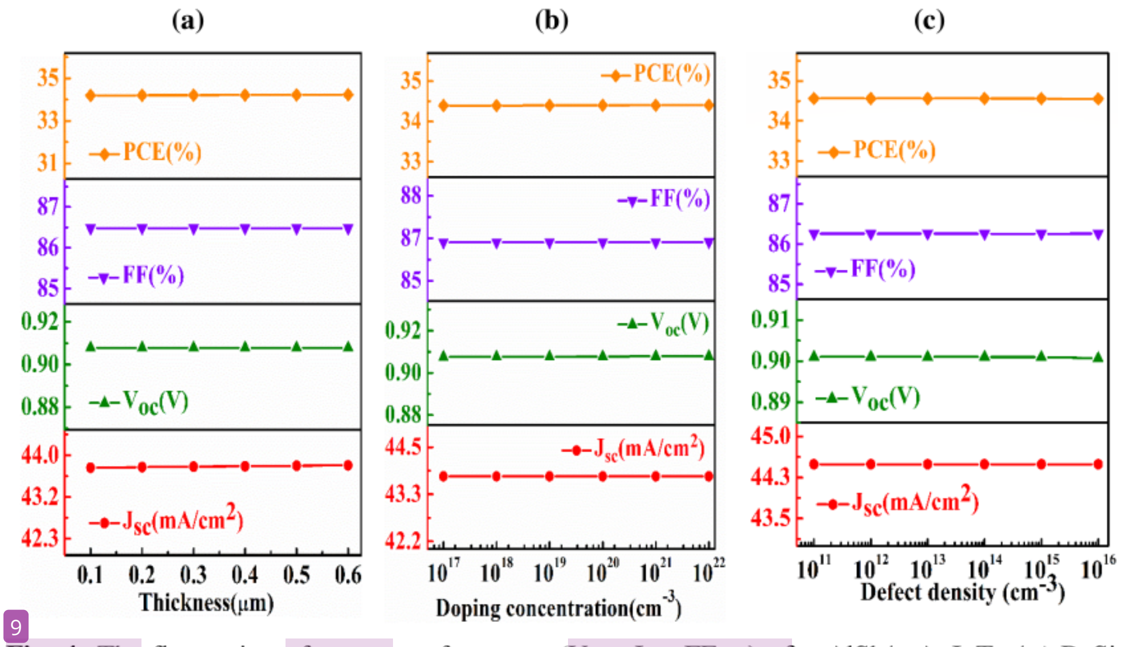

Fig. 4: The fluctuation of output performance (V_{OC}, J_{SC}, FF, η) of *n*-AlSb/*p*-AgInTe$_2$/*p*$^+$-BaSi$_2$ device as a function of (a) thickness, (b) carrier and (c) defect density of BaSi$_2$ back surface layer.

3.5 Impact of resistances on device performance

The photovoltaic performance of a cell massively influenced by the series and shunt resistances of the device. The sources of these resistances are the attachment among various active layers, metal connections, and defects related to fabrication [60-61]. The series resistance is liable for causing a drop of voltage across the body of the PV device, whereas the shunt resistance makes a short path in the device for the current when the applied voltage across the cell is zero.

Fig. 5(a) shows the modulation of output parameters with the switching of series resistance. It is perceived that both the V_{OC} and J_{SC} are less sensitive to the variation of series resistance but both the FF and efficiency are highly sensitive to the change of series resistance. This is because the improvement of series resistance decreases the FF tremendously.

Fig. 5(b) sketches the alteration of output parameters of the *n*-AlSb/*p*-AgInTe$_2$/*p*$^+$-BaSi$_2$ DH PV device as a function of shunt resistance. Except J_{SC}, all the output parameters show a positive

change with the improvement of shunt resistance up to a value of 1.5 kΩ/cm^2 and then they stay at a constant value. In other words, with the decrement of the shunt resistance the performance parameters delineate a negative impression. Thus the highest value of PCE should be recorded with the lowest value of series resistance and the highest value of shunt resistance.

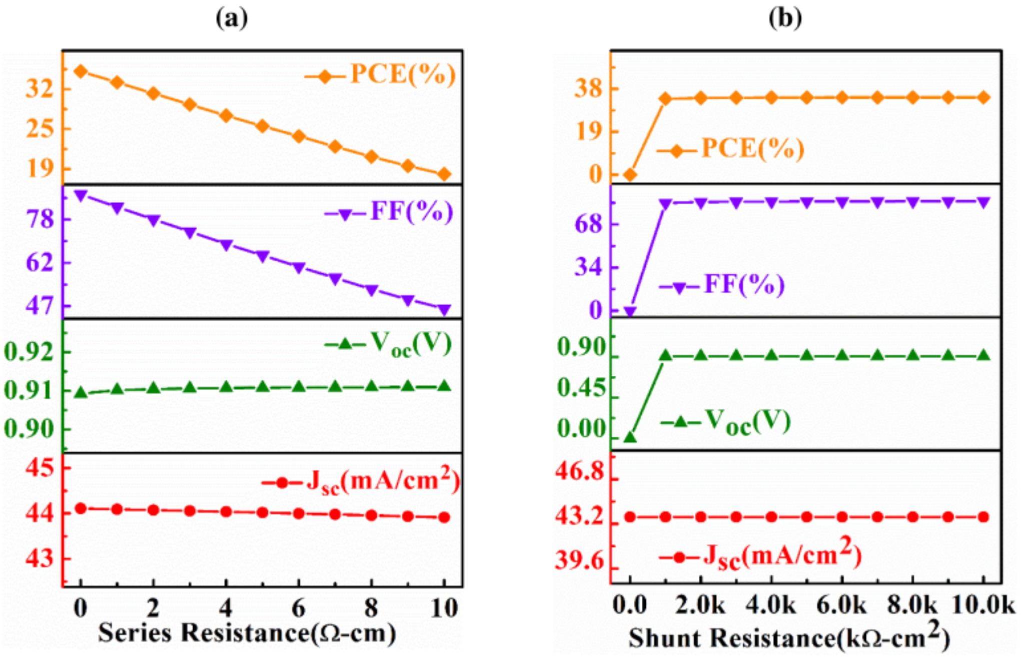

Fig. 5: The impact of (a) series and (b) shunt resistances on *n*-AlSb/*p*-$AgInTe_2$/*p*$^+$-$BaSi_2$ solar cell.

3.6 QE with and without $BaSi_2$ BSF layer

The ratio between the total aggregated charges to the quantity of alit photons is denoted as the quantum efficiency (QE) of a photovoltaic cell. We get the maximum QE of 100% when all the incident photons are converted into electric charges. We measure the quantum efficiency as a function of wavelength [53, 57]. In Fig. 6(a), the quantum efficiency has been depicted in regard to the fluctuation of wavelength with deferent thicknesses of the absorber layer. This graph shows that the quantum efficiency escalates with the rising of the absorber layer thickness. The reason behind this is the absorption of photons enhanced with a wider absorber layer which also makes more electron-hole pairs resulting higher J_{SC}.

Fig. 6(b) displays the undulation of quantum efficiency with different thickness of the $BaSi_2$ BSF layer. No variation of quantum efficiency is recorded with the seesaw of the width of the BSF layer just because the thickness of back surface field (BSF) is not creating the barrier for charge carriers. The BSF helps to confine the minority carriers generated at the surface of the PV cell. If the BSF is too thin, the minority carriers will diffuse out of the solar cell before they can be collected by the electrodes. If the BSF is too thick, the minority carriers will have a difficult time reaching the electrodes. The thickness of the BSF is therefore critical to the performance of the solar cell.

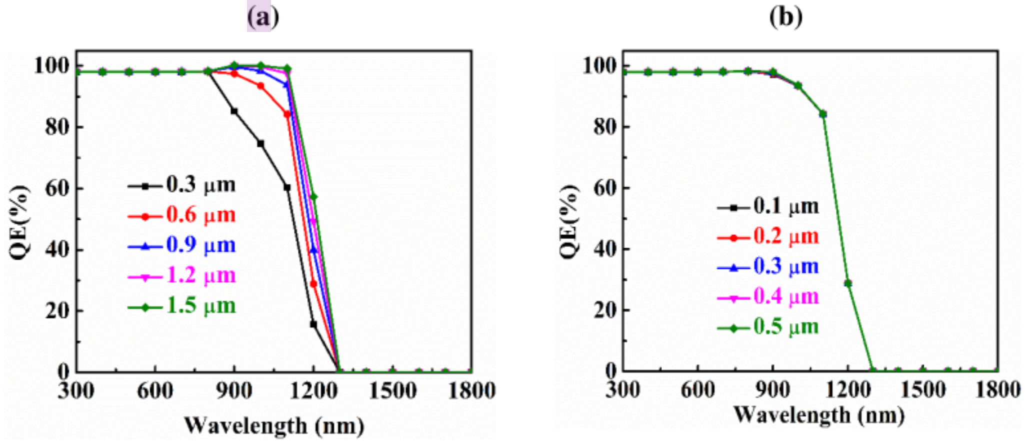

Fig. 6: Changing of quantum efficiency as a function of wavelength for (a) AIT absorber and (b) $BaSi_2$ BSF layer at various thicknesses.

3.7 Overall output of *n*-AlSb/*p*-AIT/p^+-$BaSi_2$ photovoltaic cell

Herein, the contribution of the *n*-AlSb/*p*-AIT/p^+-$BaSi_2$ photovoltaic cell has been analyzed. Fig. 7 shows the current (J)-voltage (V) graphs of the *n*-AlSb/*p*-AIT heterojunction and *n*-AlSb/*p*-AIT/p^+-$BaSi_2$ double-heterojunction PV devices with fine-tune structures. The optimal thicknesses of AlSb window, AIT absorber and $BaSi_2$ semiconductors are 0.2, 0.6, and 0.2 μm, orderly. The doping concentration of the same layers are 10^{17}, 10^{20} and 10^{20} cm^{-3}, accordingly. However, the defect densities are fixed at 10^{14}, 10^{13} and 10^{14} cm^{-3} for window, absorber and BSF layers, respectively. It is visualized from the figure that the *n*-AlSb/*p*-AIT cell architecture attains output parameters J_{SC}=43.72 mA/cm^2, V_{OC}=0.80 V, FF=85.37% and efficiency=30.03%. Whereas, with the inclusion of $BaSi_2$ layer, the *n*-AlSb/*p*-AIT heterojunction device turns into *n*-AlSb/*p*-AIT/p^+-

$BaSi_2$ PV device and the there is an improvement on the output parameters. The improved output parameters are J_{SC}=43.75 mA/cm^2, V_{OC}=0.90 V, FF=86.42% and efficiency=34.32%. The J_{SC} experiences only a meager change as all the alit photons are absorbed in AIT layer before reaching the relatively wide bandgap $BaSi_2$ BSF layer. However, there is a noticeable rise in the value of V_{OC} as the cause of the improvement of supreme built-in voltage at the *n*-AlSb/*p*-AIT and *p*-AIT/p^+-$BaSi_2$ heterojunctions. An improvement in the efficiency is also noticed and the reason behind this is the advancement of the value of V_{OC}.

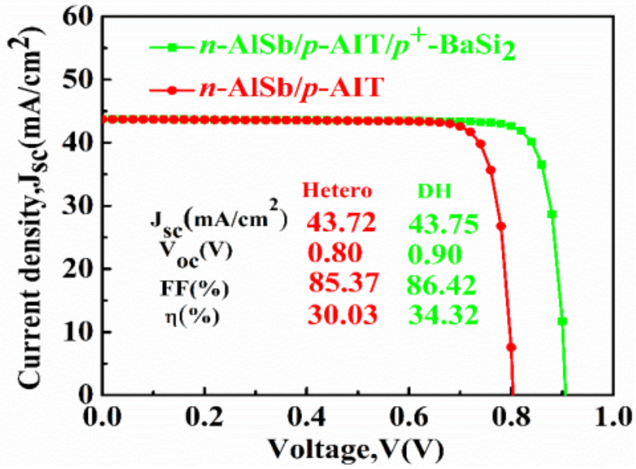

Fig. 7: The Current (J)-Voltage (V) characteristics of the AIT solar cell including and excluding $BaSi_2$ BSF layer.

However, in order to validate the potential of $BaSi_2$ BSF layer, $CuInSe_2$ semiconductor based CdS/*p*-$CuInSe_2$/p^+-$BaSi_2$ solar cell has been considered. $CuInSe_2$ (CIS) is an I-III-VI group material like AIT with an optical bandgap of 1.04 eV which is close to that of AIT (1.03 eV) [62]. CIS based ZnO/*n*-CdS/*p*-CIS with Mo metal as anode shows an experimental efficiency of ~15% with a V_{OC} of 0.513 V, J_{SC}=40.40 mA/cm^2 and FF=71.6% [63]. The same structure has been used in SCAPS that produces an efficiency of 16.39% with V_{OC}=0.526 V, J_{SC}=39.65 mA/cm^2, and FF=78.58% which is almost consistent with the experimental result. Then, $BaSi_2$ BSF layer has been added in the structure with the physical parameters shown in Table 1. The ZnO/*n*-CdS/*p*-CIS/p^+-$BaSi_2$ device with Mo anode provides a PCE of 21.65% with a V_{OC}=0.624 V, J_{SC}=45.25 mA/cm^2 and FF=76.06%. Therefore, it can be concluded that as $BaSi_2$ BSF layer shows potential

in $CuInSe_2$ based ZnO/n-CdS/p-$CuInSe_2$/p$^+$-$BaSi_2$ device, it will also have high impact on *n*-AlSb/*p*-$AgInTe_2$/*p*$^+$-$BaSi_2$ dual-heterojunction thin film photovoltaic cells.

4. Conclusion

In this effort, we have explored the operation of a photovoltaic device based on $AgInTe_2$ ternary chalcopyrite semiconducting material. $AgInTe_2$ as the absorber layer, AlSb as the window layer, and $BaSi_2$ as the back surface field layer have been chosen for the device structure. Best performance has been attained by taking the absorber width of 0.6 μm, BSF layer thickness of 0.2 μm and the window layer thickness of 0.2 μm. The excellent values of performance parameters which have been obtained are J_{SC}=43.75 mA/cm^2, V_{OC}=0.90 V, FF=86.42% and efficiency=34.32%. Undoubtedly, these numbers are rich in the present days. Further research in this device may attain more fruitful results in future. Hopefully, these outcomes instigate the potential of highly effective AIT-based *n*-AlSb/*p*-AIT/*p*$^+$-$BaSi_2$ photovoltaic cell to combat the world energy crisis.

Acknowledgements

The authors are indebted to Prof. Dr. Marc Burgelman, University of Gent, Belgium, for imparting SCAPS simulation software.

Corresponding author:

*E-mail: jak_apee@ru.ac.bd (Jaker Hossain).

Notes: The authors declare no competing financial interest.

Data Availability: Simulation details and data are available from authors upon reasonable request.

References

[1] S. Styring, Artificial photosynthesis for solar fuels, Faraday Discuss. 155 (2012) 357-376.

[2] T. K. Nideep, M. Ramya, M. Kailasnath, An investigation on the photovoltaic performance of quantum dot solar cells sensitized by CdTe, CdSe and CdS having comparable size, Superlattices Microstruct. 141 (2020) 106477.

[3] V. Steinmann, R. E. Brandt, T. Buonassisi, Photovoltaics: non-cubic solar cell materials, Nat. Photonics 9 (2015) 355–357.

[4] R. M. Swanson, Developments in silicon solar cells, Proceedings of IEEE Electron Devices Meeting, Washington, DC, 10–12 December 2007 (IEEE, 2007), pp. 359–362.

[5] D. Sarti, R. Einhaus, Silicon feedstock for the multi-crystalline photovoltaic industry, Sol. Energy Mater. Solar Cells 72 (2002) 27-40.

[6] A. G. Aberle, Surface passivation of crystalline silicon solar cells: a review, Prog. Photovoltaics 8 (2000) 473-487.

[7] F. Dross, J. Robbelein, B. Vandevelde, E. Van Kerschaver, I. Gordon, G. Beaucarne, J. Poortman, Stress-induced large-area lift-off of crystalline Si films, Appl. Phys. A 89 (2007) 149-152.

[8] T. Mishima, M. Taguchi, H. Sakata, and E. Maruyama, Development status of high-efficiency HIT solar cells, Sol. Energy Mater. Solar Cells 95 (2011) 18-21.

[9] K. Yoshikawa, H. Kawasaki, W. Yoshida, T. Irie, K. Konishi, K. Nakano, T. Uto, D. Adachi, M. Kanematsu, H. Uzu, K. Yamamoto, Silicon heterojunction solar cell with interdigitated back contacts for a photoconversion efficiency over 26%, Nature Energy 2 (2017) 17032.

[10] A. Richter, M. Hermle, S. W. Glunz, Reassessment of the limiting efficiency for crystalline silicon solar cells, IEEE J. Photovoltaics 3 (2013) 1184–1191.

[11] I. Almansouri, A. Ho-Baillie, S. P. Bremner, M. A. Green, Supercharging Silicon Solar Cell Performance by Means of Multijunction Concept, IEEE J. Photovoltaics 5 (2015) 968–976.

[12] A. D. Vos, Detailed balance limit of the efficiency of tandem solar cells, J. Phys. D Appl. Phys. 13 (1980) 839–846.

[13] A. S. Brown, M. A. Green, Detailed balance limit for the series constrained two terminal tandem solar cell, Physica E 14 (2002) 96–100.

[14] I. V. Bodnar, V. F. Gremenok, K. Bente, Th. Doering, W. Schmitz, Optical Properties of $AgInTe_2$ Films Prepared by Pulsed Laser Deposition, Phys. Status Solidi (a), 175 (1999) 607-613.

[15] A.S. Verma, Thermal properties of chalcopyrite semiconductors, Phil. Mag. 89 (2009) 183-193.

[16] A. V. Kopytov , A. V. Kosobutsky, Thermodynamic and elastic properties of $AgInSe_2$ and $AgInTe_2$, Physics of the Solid State 52 (2010) 1359–1361.

[17] A. Jagomagi, J. Krustok, J. Raudoja, M. Grossberg, I. Oja, M. Krunks, M. Danilson, Photoluminescence and Raman spectroscopy of polycrystalline $AgInTe_2$, Thin Solid Films 480–481 (2005) 246-249.

[18] Y. Zhang, Bandgap nature of chalcopyrite $ZnXP_2$ (X=Si, Ge, Sn), Comp. Mater. Sci. 133 (2017) 152–158.

[19] S. Bagci, B. G. Yalcin, H.A.R. Aliabad, S. Duman, B. Salmankurt, Structural, electronic, optical, vibrational and transport properties of CuBX2 (X = S, Se, Te) chalcopyrites, RSC Adv. 6 (2016) 59527–59540.

[20] V. K. Gudelli, V. Kanchana, G. Vaitheeswaran, $CuAlTe_2$: A promising bulk thermoelectric material, J. Alloys Compd. 648 (2015) 958–965.

[21] S. Sharma, A. S. Verma, R. Bhandari, S. Kumari, V.K. Jindal, Ab initiostudies of structural, electronic, optical, elastic and thermal properties of Ag-chalcopyrites ($AgAlX_2$: X = S, Se), Mater. Sci. Semicond. Proc. 26 (2014) 187–198.

[22] P. Pichanusakorn, P. Bandaru, Nanostructured thermoelectrics, Mater. Sci. Eng. R 67 (2010) 19–63.

[23] A. Charoenphakdee, K. Kurosaki, H. Muta, M. Uno, S. Yamanaka, Thermal conductivity of the ternary compounds: $AgMTe_2$ and AgM_5Te_8 (M=Ga or In), Mater. Trans. 50 (2009) 1603–1606.

[24] D. C. Nguyen, S. Ito, Narrow Band Gap $AgInTe_2$ Solar Cells Fabricated by Printing Method, Energy Science and Technology, 4 (2012), 1-5. http://dx.doi.org/10.3968/j.est.1923847920120402.536

[25 A. Uzum, N. Takahashi, S. Ito, Narrow Bandgap Solar Cells Using AgInTe2, 29th European Photovoltaic Solar Energy Conference and Exhibition, 22 - 26 September 2014, pp-356 – 358. https://doi.org/10.4229/EUPVSEC20142014-1BV.7.75

[26] K. L. Chopra, P. D. Paulson, V. Dutta, Thin-film solar cells: An overview, Progress in Photovoltaics 12 (2004) 69–92.

[27] M. K. S. B. Rafiq, N. Amin, H. F. Alharbi, M. Luqman, A. Ayob, Y. S. Alharthi, N. H. Alharthi, B. Bais, M. Akhtaruzzaman, WS_2: A New Window Layer Material for Solar Cell Application, Sci. Rep. 10 (2020) 771.

[28] D. Lilhare, A. Khare, Development of chalcogenide solar cells: Importance of CdS window layer, Opto-Electronics Review 28 (2020) 43-63.

[29] J. He, L. Wu, L. Feng, J. Zheng, J. Zhang, W. Li, B. Li, Y. Cai, Structural, electrical and optical properties of annealed Al/Sb multiayer films, Sol. Energy Mater. Solar cells 95 (2011) 369-372.

[30] K. Seeger, E. Schonherr, Microwave dielectric constant of aluminium antimonide, Semicond. Sci. Technol. 6 (1991) 301.

[31] F. F. Yao, Z. Lei, L. H. Feng, J. Q. Zhang, W. Li, L. L. Wu, W. Cai, Y. P. Cai, J. G. Zheng, B. Li, Preparation of AlSb polycrystalline thin films by co-evaporation, Chin. J. Semicond. 27 (2006) 1578.

[32] A. Hemani, D. Benmoussa, A. Nouri, H. Khachab, Effect of the FSF and BSF layers on the performances of the GaAS solar cell, J. Ovonic Research 13 (2017) 307-314.

[33] M. M. A. Moon, M. H. Ali, M. F. Rahman, A. Kuddus, J. Hossain, A. B. M. Ismail, Investigation of thin-film p-$BaSi_2$/n-CdS heterostructure towards semiconducting silicide based high efficiency solar cell, Phys. Scr. 95 (2020) 035506.

[34] K. Morita, Y. Inomata, T. Suemasu, Optical and electrical properties of semiconducting $BaSi_2$ thin films on Si substrates grown by molecular beam epitaxy, Thin Solid Films 508 (2006) 363.

[35] F. J. Zhao, Q. Xie, Q. Chen, C. H. Yang, First-principles calculations on the electronic structure and optical properties of $BaSi_2$, Science in China Series G: Physics, Mechanics and Astronomy 52 (2009) 580–586.

[36] K. O. Hara, Y. Nakagawa, T. Suemasu, N. Usami, Simple vacuum evaporation route to $BaSi_2$ thin films for solar cell applications, Procedia Engineering 141 (2016) 27-31.

[37] T. Deng, T. Sato, Z. Xu, R. Takabe, S. Yachi, Y. Yamashita, K. Toko, T. Suemasu, p-$BaSi_2$/n-Si heterojunction solar cells on Si(001) with conversion efficiency approaching 10%: comparison with Si(111), Appl. Phys. Express 11 (2018) 062301.

[38] W. Du, R. Takabe, M. Baba, H. Takeuchi, K.O. Hara, K. Toko, N. Usami, T. Suemasu, Formation of $BaSi_2$ heterojunction solar cells using transparent MoOx hole transport layers, Appl. Phys. Lett. 106 (2015) 122104.

[39] D. Fomin, V. Dubov, K. Galkin, N. Galkin, R. Batalov, V. Shustov, Formation and properties of crystalline BaSi2 thin films obtained by solid phase epitaxy on Si(111), JJAP Conf. Proc. 5 (2017) 011203.

[40] T. Yoneyama, A. Okada, M. Suzuno, T. Shibutami, K. Matsumaru, Formation of polycrystalline $BaSi_2$ films by radio-frequency magnetron sputtering for thin-film solar cell applications, Thin Solid Films 534 (2013) 116–119.

[41] T. Nakamura, T. Suemasu, K.-I. Takakura, F. Hasegawa, Investigation of the energy band structure of orthorhombic $BaSi_2$ by optical and electrical measurements and theoretical calculations, Appl. Phys. Let. 81 (2002) 1032–1034.

[42] Q. Ma, H. Kyureghian, J. Banninga, N. Ianno, Thin-film AlSb for use as a photovoltaic absorber material, Materials Research Society, Symposium E 1670 (2014) 10-02.

[43] P. Tang, W. Wang, B. Li, L. Feng, G. Zeng, The properties of Zn-doped AlSb thin films prepared by pulsed laser deposition, Coatings 9 (2019) 136.

[44] A. El-Korashy, M. A. Abdel-Rahim, H. El-Zahed, Optical absorption studies on $AgInSe_2$ and $AgInTe_2$ thin films, Thin Solid Films 338 (1999) 207–212.

[45] N. Benseddik, B. Belkacemi, F. Boukabrine, K. Ameur, H. Mazari, A. Boumesjed, N. Benyahya, Z. Benamara, Numerical study of $AgInTe_2$ solar cells using SCAPS. Adv. Mater. Process. Technol. 2020. https://doi.org/10.1080/2374068X.2020.1833401.

[46] R. Vismara, O. Isabella, and M. Zeman, Organometallic halide perovskite/barium di-silicide thin-film doublejunction solar cells Proc. SPIE, Photonics for Solar Energy Systems VI 9898 (Brussels, Belgium, April 29, 2016) 98980J

[47] L. Chen, H. Chen, Q. Deng, G. Wang, S. Wang, Numerical simulation of planar $BaSi_2$ based Schottky junction solar cells toward high efficiency, Solid-State Electron. 149 (2018) 46-51.

[48] J. Hossain, M. Rahman, M. M. A. Moon, B. K. Mondal, M. F. Rahman, M. H. K. Rubel, Guidelines for a highly efficient CuI/n-Si heterojunction solar cell, Eng. Res. Express 2 (2020) 045019.

[49] T. Ouslimane, L. Et-taya, L. Elmaimouni, A. Benami, Impact of absorber layer thickness, defect density, and operating temperature on the performance of $MAPbI_3$ solar cells based on ZnO electron transporting material, Heliyon 7 (2021) e06379.

[50] D. K .Shah, K.C. Devendra, M. Muddassir, M. S. Akhtar, C. Y. Kim, O.-B. Yang, A simulation approach for investigating the performances of cadmium telluride solar cells using doping concentrations, carrier lifetimes, thickness of layers, and band gaps, Solar Energy

216 (2021) 259-265.

[51] C. S. Jiang, M. Yang, Y. Zhou, B. To, S. U. Nanayakkara, J. M. Luther, W. Zhou, J. J. Berry, J. Lagemaat, N. P. Padture, K. Zhu, M. M. Al-Jassim, Carrier separation and transport in perovskite solar cells studied by nanometre-scale profiling of electrical potential, Nat. Commun. 6 (2015) 8397 .

[52] B. K. Mondal, S. K. Mostaque, J. Hossain, Theoretical insights into a high-efficiency Sb_2Se_3-based dual-heterojunction solar cell, Heliyon 8 (2022) e09120.

[53] M. M. A. Moon, M. H. Ali, M. F. Rahman, J. Hossain, A. B. M. Ismail, Design and simulation of $FeSi_2$-based novel heterojunction solar cells for harnessing visible and near-infrared light, Phys. Status Solidi a 217 (2020) 1900921.

[54] A. Kuddus, Ismail, A. B. M. Ismail, J. Hossain, Design of a highly efficient CdTe-based dual-heterojunction solar cell with 44% predicted efficiency, Solar Energy 221 (2021) 488-501.

[55] J. Hossain, Design and simulation of double-heterojunction solar cells based on Si and GaAs wafers, J. Phys. Commun. 5 (2021) 085008.

[56] J. Hossain, B. K. Mondal and S. K. Mostaque, Design of a highly efficient FeS_2-based dual-heterojunction thin film solar cell, Int. J. Green Energy 14 (2022) 1531–1542.

[57] J. Hossain, M. M. A. Moon, B. K. Mondal, M. A. Halim, Design guidelines for a highly efficient high-purity germanium (HPGe)-based double-heterojunction solar cell, Opt. Laser Technol. 143 (2021) 107306.

[58] Y. H. Khattak, F. Baig, H. Toura, S. Beg, B. M. Soucase, Efficiency enhancement of Cu_2BaSnS_4 experimental thin-film solar cell by device modeling, *J. Mater. Sci*. 54 (2019) 14787–14796.

[59] Ferdiansjah, Faridah, K. T. Mularso, Analysis of Back Surface Field (BSF) Performance in P-Type And N-Type Monocrystalline Silicon Wafer, E3S Web of Conferences 43 (2018) 01006.

[60] S. Ahmmed, A. Aktar, J. Hossain, A. B. M. Ismail, Enhancing the open circuit voltage of the SnS based heterojunction solar cell using NiO HTL Sol. Energy 207 (2020) 693–702.

[61] S. R. I. Biplab, M. H. Ali, M. M. A. Moon, M. F. Pervez, M. F. Rahman, J. Hossain, Performance enhancement of CIGS-based solar cells by incorporating an ultrathin $BaSi_2$ BSF layer J. Comput. Electron. 19 (2019) 342–52.

[62] B. K. Mondal, S. K. Mostaque, and J. Hossain, Unraveling the effects of a GeSe BSF layer on the performance of a $CuInSe_2$ thin film solar cell: a computational analysis, Optics Continuum 2 (2023) 428-440.

[63] L. Stolt, J. Hedström, J. Kessler, M. Ruckh, K. O. Velthaus, and H. W. Schock, "ZnO/CdS/$CuInSe_2$ thin-film solar cells with improved performance," Appl. Phys. Lett. **62**(6), 597 (1998).

Numerical studies on a ternary AgInTe2 chalcopyrite thin film solar cell

ORIGINALITY REPORT

26%
SIMILARITY INDEX

PRIMARY SOURCES

1	www.researchgate.net<br>Internet	165 words — 3%
2	arxiv.org<br>Internet	79 words — 2%
3	Jaker Hossain, Bipanko Kumar Mondal, Shaikh Khaled Mostaque. "Computational investigation on the photovoltaic performance of an efficient GeSe-based dual-heterojunction thin film solar cell", Semiconductor Science and Technology, 2021<br>Crossref	64 words — 1%
4	iopscience.iop.org<br>Internet	60 words — 1%
5	Ahnaf Tahmid Abir, Arifuzzaman Joy, Bipanko Kumar Mondal, Jaker Hossain. "Numerical prediction on the photovoltaic performance of CZTS‐based thin film solar cell", Nano Select, 2022<br>Crossref	54 words — 1%
6	proxy.osapublishing.org<br>Internet	46 words — 1%
7	Md Sabuj Hossen, Ahnaf Tahmid Abir, Jaker Hossain. " Design of an efficient AgInSe chalcopyrite‐based	43 words — 1%

heterojunction thin film solar cell ", Energy Technology, 2023
Crossref

---

8 Joyprokash Chakrabartty, Md. Aminul Islam, Sahariar Reza. "Performance analysis of highly efficient 2D/3D bilayer inverted perovskite solar cells", Solar Energy, 2021 — 41 words — 1%
Crossref

---

9 Abdul Kuddus, Abu Bakar Md. Ismail, Jaker Hossain. "Design of a highly efficient CdTe-based dual-heterojunction solar cell with 44% predicted efficiency", Solar Energy, 2021 — 40 words — 1%
Crossref

---

10 Sheikh Rashel Al Ahmed, Adil Sunny, Sabrina Rahman. "Performance enhancement of Sb2Se3 solar cell using a back surface field layer: A numerical simulation approach", Solar Energy Materials and Solar Cells, 2021 — 37 words — 1%
Crossref

---

11 Shaikh Khaled Mostaque, Bipanko Kumar Mondal, Jaker Hossain. "Numerical simulation on the impurity photovoltaic (IPV) effect in c-Si wafer-based dual-heterojunction solar cell", Materials Today Communications, 2022 — 34 words — 1%
Crossref

---

12 Jaker Hossain, Bipanko Kumar Mondal, Shaikh Khaled Mostaque. " Design of a highly efficient FeS -based dual-heterojunction thin film solar cell ", International Journal of Green Energy, 2021 — 32 words — 1%
Crossref

---

13 Sheikh Rashel Al Ahmed, Mostafizur Rahaman, Adil Sunny, Sabrina Rahman et al. "Enhancing the efficiency of Cu2Te thin-film solar cell with WS2 buffer layer: A simulation study", Optics & Laser Technology, 2023 — 24 words — < 1%
Crossref

14 www.science.gov
Internet
24 words — < 1%

15 Bipin Saha, Bipanko Kumar Mondal, Shaikh Khaled Mostaque, Mainul Hossain, Jaker Hossain. " Numerical modeling of CuSbSe -based dual-heterojunction thin film solar cell with CGS back surface layer ", AIP Advances, 2023
Crossref
21 words — < 1%

16 Shaikh Khaled Mostaque, Bipanko Kumar Mondal, Jaker Hossain. "Theoretical insight into the enhancement of longer-wavelength light absorption in silicon solar cell with multilevel impurities", Results in Optics, 2022
Crossref
18 words — < 1%

17 Md Sabuj Hossen, Ahnaf Tahmid Abir, Jaker Hossain. " Design of an Efficient AgInSe Chalcopyrite - Based Heterojunction Thin - Film Solar Cell ", Energy Technology, 2023
Crossref
17 words — < 1%

18 Md. Choyon Islam, Bipanko Kumar Mondal, Tanvir Ahmed, Md. Alamin Hossain Pappu, Shaikh Khaled Mostaque, Jaker Hossain. "Design of a highly efficient n-CdS/p-AgGaTe2/p+-SnS double-heterojunction thin film Solar cell", Engineering Research Express, 2023
Crossref
17 words — < 1%

19 digitalassets.lib.berkeley.edu
Internet
17 words — < 1%

20 www.epjap.org
Internet
17 words — < 1%

link.springer.com

21 Internet — 16 words — < 1%

22 tel.archives-ouvertes.fr
Internet — 16 words — < 1%

23 thesis.univ-biskra.dz
Internet — 16 words — < 1%

24 www.researchsquare.com
Internet — 16 words — < 1%

25 M. Atowar Rahman. "Performance analysis of WSe2-based bifacial solar cells with different electron transport and hole transport materials by SCAPS-1D", Heliyon, 2022
Crossref — 12 words — < 1%

26 N.M. Shaalan, K.O. Hara, C.T. Trinh, Y. Nakagawa, N. Usami. "Simple method for significant improvement of minority-carrier lifetime of evaporated BaSi 2 thin film by sputtered-AlO x passivation", Materials Science in Semiconductor Processing, 2018
Crossref — 11 words — < 1%

27 api-depositonce.tu-berlin.de
Internet — 11 words — < 1%

28 assets.researchsquare.com
Internet — 11 words — < 1%

29 Adil Sunny, Sheikh Rashel Al Ahmed. " Numerical Simulation and Performance Evaluation of Highly Efficient Sb Se Solar Cell with SnS as Hole Transport Layer ", physica status solidi (b), 2021
Crossref — 10 words — < 1%

30 I. Chabri, Y. Benhouria, A. Oubelkacem, A. Kaiba, I. Essaoudi, A. Ainane. "Enhance stability of γ-CsSnI3-based PSCs by (γ-CsSnI3-Cs2SnI6) heterojunction", Solar Energy Materials and Solar Cells, 2023 — 10 words — < 1%
Crossref

31 Ryota Takabe, Tianguo Deng, Komomo Kodama, Yudai Yamashita, Takuma Sato, Kaoru Toko, Takashi Suemasu. "Impact of Ba to Si deposition rate ratios during molecular beam epitaxy on carrier concentration and spectral response of BaSi2 epitaxial films", Journal of Applied Physics, 2018 — 9 words — < 1%
Crossref

32 Sabrina Rahman, Sheikh Rashel Al Ahmed. "Photovoltaic performance enhancement in CdTe thin-film heterojunction solar cell with Sb2S3 as hole transport layer", Solar Energy, 2021 — 9 words — < 1%
Crossref

33 Shaikh Khaled Mostaque, Bipanko Kumar Mondal, Jaker Hossain. "Simulation approach to reach the SQ limit in CIGS-based dual-heterojunction solar cell", Optik, 2021 — 9 words — < 1%
Crossref

34 Shamim Ahmmed, Asma Aktar, Ferdous Rahman, Jaker Hossain, Abu Bakar Md. Ismail. "A numerical simulation of high efficiency CdS/CdTe based solar cell using NiO HTL and ZnO TCO", Optik, 2020 — 9 words — < 1%
Crossref

35 Shamim Ahmmed, Asma Aktar, Samia Tabassum, Md. Hafijur Rahman, Md. Ferdous Rahman, Abu Bakar Md. Ismail. "CuO based solar cell with V2O5 BSF layer: Theoretical validation of experimental data", Superlattices and Microstructures, 2021 — 9 words — < 1%

Crossref

36 digitalcommons.usf.edu
Internet
9 words — < 1%

37 discovery.ucl.ac.uk
Internet
9 words — < 1%

38 kops.uni-konstanz.de
Internet
9 words — < 1%

39 lup.lub.lu.se
Internet
9 words — < 1%

40 worldwidescience.org
Internet
9 words — < 1%

41 Akito Sasaki, Yoshinori Kataoka, Katsuaki Aoki, Syuichi Saito, Kumpei Kobayashi, Takahiro Ito, Kuniyuki Kakushima, Hiroshi Iwai. "Power generation characteristics of Schottky-type solar cells fabricated using barium silicide", Japanese Journal of Applied Physics, 2015
Crossref
8 words — < 1%

42 Hafeez Anwar, Syed Zulqarnain Haider, Mingqing Wang. "A comprehensive device modeling of perovskite solar cell with inorganic copper iodide as hole transport material", Semiconductor Science and Technology, 2018
Crossref
8 words — < 1%

43 Md Dulal Haque, Md Hasan Ali, Md Ferdous Rahman, Abu Zafor Md Touhidul Islam. "Numerical analysis for the efficiency enhancement of MoS2 solar cell: A simulation approach by SCAP-1D", Optical Materials, 2022
Crossref
8 words — < 1%

44 Nabeel H. Alharthi, Saud M. Almotairy, Hamad F. Alharbi, M. Shahinuzzaman, Monis Luqman, K. Sobayel. "Investigation on Structural and Opto-electronic Properties of Substitutional Sn Doped WS2 by Co-sputtering Technique", Journal of Materials Research and Technology, 2021 — 8 words — < 1%
Crossref

45 Nadia Benseddik, Benyamin Belkacemi, Fouzia Boukabrine, Kheira Ameur et al. " Numerical study of AgInTe solar cells using SCAPS ", Advances in Materials and Processing Technologies, 2020 — 8 words — < 1%
Crossref

46 Neelima Singh, Alpana Agarwal, Mohit Agarwal. "Numerical simulation of highly efficient lead-free perovskite layers for the application of all-perovskite multi-junction solar cell", Superlattices and Microstructures, 2021 — 8 words — < 1%
Crossref

47 Premshila Kumari, Urvashi Punia, Deepak Sharma, Avritti Srivastava, Sanjay K. Srivastava. "Enhanced Photovoltaic Performance of PEDOT:PSS/Si Heterojunction Solar Cell with ZnO BSF Layer: A Simulation Study using SCAPS-1D", Silicon, 2022 — 8 words — < 1%
Crossref

48 Sakib Mohammad, Md. Noumil Tousif, A. A. Ferdous, Md Ashraful Hoque, Mohammad Wahidur Rahman. "Numerical analysis of czts solar cell with in2s3 buffer layer: a study of a czts based thin film solar cell, concerning the effects of several parameters on its electrical performance", 2017 IEEE International Conference on Smart Grid and Smart Cities (ICSGSC), 2017 — 8 words — < 1%
Crossref

cyberleninka.org

49 Internet

8 words — < 1%

50 downloads.hindawi.com
Internet

8 words — < 1%

51 openresearch-repository.anu.edu.au
Internet

8 words — < 1%

52 repository.kaust.edu.sa
Internet

8 words — < 1%

53 riunet.upv.es
Internet

8 words — < 1%

54 upcommons.upc.edu
Internet

8 words — < 1%

55 vdoc.pub
Internet

8 words — < 1%

56 www.lmpv.nl
Internet

8 words — < 1%

57 L.I. Nykyruy, R.S. Yavorskyi, Z.R. Zapukhlyak, G. Wisz, P. Potera. "Evaluation of CdS/CdTe thin film solar cells: SCAPS thickness simulation and analysis of optical properties", Optical Materials, 2019
Crossref

7 words — < 1%

58 Li, Jiakai. "Electrical, Optical and High Frequency Performance Improvement for Type-II Superlattices Based Photodetectors", Northwestern University, 2023
ProQuest

7 words — < 1%

59 Yao Yao, Xiaoyu Xu, Xiumei Zhang, Haiping Zhou, Xiaofeng Gu, Shaoqing Xiao. "Enhanced efficiency in bifacial HIT solar cells by gradient doping with AFORS-HET simulation", Materials Science in Semiconductor Processing, 2018 — 7 words — < 1%
Crossref

60 oz, . "Principles and Systems in Light-Induced Energy Conversion", Photoelectrochemical Solar Conversion Systems Molecular and Electronic Aspects, 2012. — 7 words — < 1%
Crossref

61 Djicknoum Diouf. "Two-Dimensional Simulations of Interdigitated Back Contact Silicon Heterojunctions Solar Cells", Engineering Materials, 2012 — 6 words — < 1%
Crossref

62 G. D. K. Mahanama *, H. S. Reehal. "Dark and illuminated characteristics of crystalline silicon solar cells with ECR plasma CVD deposited emitters", International Journal of Electronics, 2005 — 6 words — < 1%
Crossref

63 Md. Choyon Islam, Bipanko Kumar Mondal, Tanvir Ahmed, Md. Alamin Hossain Pappu, Shaikh Khaled Mostaque, Jaker Hossain. " Design of a highly efficient n-CdS/p-AgGaTe /p+-SnS double-heterojunction thin film solar cell ", Engineering Research Express, 2023 — 6 words — < 1%
Crossref

64 Md. Hasan Ali, Md. Dulal Haque, Md. Mahabub Hossain, Abu Zafor Md. Touhidul I. "Improving the efficiency of ZnTe based heterojunction solar cell with In2Te3 BSF layer", Research Square Platform LLC, 2023 — 6 words — < 1%
Crossref Posted Content

65	Ubaid ur Rehman, N. Almousa, Kashaf ul Sahar, Arslan Ashfaq et al. " Optimizing the efficiency of lead free Cs TiI - based double halide perovskite solar cells using SCAPS - 1D ", Energy Technology, 2023<br>Crossref	6 words — < 1%
66	www.utupub.fi<br>Internet	6 words — < 1%

EXCLUDE QUOTES	OFF	EXCLUDE SOURCES	OFF
EXCLUDE BIBLIOGRAPHY	ON	EXCLUDE MATCHES	OFF
